# The Micro-Immunotherapy Medicine 2LPAPI^®^ Displays Immune-Modulatory Effects in a Model of Human Papillomavirus Type-16 L1-Protein Capsid-Treated Human Peripheral Blood Mononuclear Cells and Antiproliferative Effects in a Model of Cervical Cancer Cells

**DOI:** 10.3390/cancers16071421

**Published:** 2024-04-05

**Authors:** Camille Jacques, Flora Marchand, Mathias Chatelais, Virginie Albinet, Claire Coustal, Ilaria Floris

**Affiliations:** 1 Preclinical Research Department, Labo’Life France, Pescalis-Les Magnys, 79320 Moncoutant-sur-Sevre, France; ilaria.floris@labolife.com; 2 ProfileHIT, 7 rue du Buisson, 44680 Sainte-Pazanne, France; flora.marchand@profile-hit.com (F.M.); mathias.chatelais@profile-hit.com (M.C.); 3 Imavita S.A.S., Canal Biotech 1&2, 3 rue des Satellites, Parc Technologique du Canal, 31400 Toulouse, France; virginie.albinet@imavita.com (V.A.); claire.coustal@imavita.com (C.C.)

**Keywords:** micro-immunotherapy, cytokines, papillomavirus, cervical cancer, HeLa, immune response, lymphocytes, immune-regulator

## Abstract

**Simple Summary:**

The human papillomavirus (HPV), a major carcinogenic pathogen, can cause cervical cancer through persistent infection. The immune system typically fights off the virus, albeit long-term activation may promote carcinogenesis. The micro-immunotherapy medicine 2LPAPI^®^ holds promise for aiding viral clearance and mitigating cervical cancer risk, and our research aimed to examine the effects of this medicine in vitro. We focused our investigations on the two most prevalent genotypes of HPV causing persistent infection, HPV-16 and HPV-18. We found that 2LPAPI^®^ boosted the secretion of IL-6, IFN-γ, and IP-10 in human immune cells when exposed to HPV-16 proteins, suggesting enhanced defensive responses to HPV-16. Some of the active substances curtailed T-cell proliferation and activity and displayed antiproliferative properties on HPV-18 positive cervical cancer-derived HeLa cells in nutrient-restricted conditions. These results unveil 2LPAPI^®^’s potential dual role: immunomodulation for HPV-16-affected immune cells and antiproliferative activity against a model of HPV-18-positive-cervical cancer cells.

**Abstract:**

Human papillomavirus (HPV) is the second most common infectious agent causing cancer. Persistent infection with high-risk (HR)-HPV can lead to cervical intra-epithelial neoplasia and cervical carcinomas (CC). While host immune response is necessary for viral clearance, chronic immune activation contributes to a low-grade inflammation that can ultimately lead to carcinogenesis. The micro-immunotherapy medicine (MIM) 2LPAPI^®^ could be a valuable tool to manage the clearance of the virus and reduce the risk of developing CC. In this in vitro study, we aimed to investigate its mode of action. We showed that actives from the MIM increased the IL-6, IFN-γ, and IP-10 secretion in human peripheral blood mononuclear cells (PBMCs) exposed to peptides derived from the HPV-16 capsid (HPV16_(L1)_). This could reflect an increase in the immune activity toward HPV-16. At the same time, some active substances reduced the lympho-proliferation and the expression of T-cell activation markers. Finally, some of the MIM actives displayed antiproliferative effects in CC-derived HeLa cells under serum-starvation conditions. Altogether, this body of data highlighted for the first time the dual effect of MIM in the framework of HR-HPV infections as a potential (i) immune modulator of HPV16_(L1)_-treated PBMCs and (ii) antiproliferative agent of HPV-positive CC cells.

## 1. Introduction

Human papillomavirus (HPV) is a small, non-enveloped, cyclic double-stranded deoxyribonucleic acid (DNA), a sexually transmissible virus characterized by its specific tropism for epithelial cells. In 2018, in the United States, it was estimated that the number of persons infected was 42.5 million among 15- to 59-year-olds, and about 6.1 million women acquired a new disease-associated HPV-type infection within the same age group that year [1]. Recognized as the second most common infectious agent causing cancer [2], persistent infection with HPV is associated with cervical intra-epithelial neoplasia (CIN) and cervical carcinomas (CC). Moreover, HPV is also attributable to non-malignant anogenital warts as well as other less prevalent anogenital cancers and oropharyngeal cancers. Of note, and while the causative role of HPV has to be confirmed, HPV infection was even found to be associated with the occurrence of adenocarcinoma of the lung as well [3]. 

Amongst the 200 existing HPV genotypes, and according to the International Agency for Research on Cancer (IARC), fourteen of them, including the HPV-16 and HPV-18, are considered high-risk (HR) genotypes due to their carcinogenic properties [4,5,6]. Even if the complete mechanisms participating in viral persistence are not yet fully elucidated, it is established that HPV type, women’s age, sexual behavior, and parity influence the time-to-viral-clearance [7]. A worldwide meta-analysis of persistence patterns estimated that HPV-infected women tend to resolve the infection within 6 to 12 months [8]. Another recent meta-analysis revealed that the overall persistent HPV infection prevalence in the world is 29.37%, and by genotype, HPV-16 displayed the highest global and European prevalence (35% and 40%, respectively) [9]. The host cell-mediated immune response is crucial in clearing and resolving HPV infections. This theory is sustained by histopathological analysis that revealed the presence of macrophages and T-cell infiltrates in regressing genital warts [10]. The carcinoma stage is preceded by three premalignant phases, also named CIN1, CIN2, and CIN3. In early CIN1 and CIN2 lesions, the viral genome is present in the form of episome, while in pre-cancer CIN3 and cancer tissues, the viral genome was found integrated into the host genome [11]. This suggests that the major event causing cervical carcinogenesis is the HR-HPV genome integration. Following the integration, the infected cells increase the expression of the two key viral oncogenes, E6 and E7, both involved in the neoplastic initiation and the progression of CC. Indeed, E6 and E7 mediate malignant transformation through the degradation of p53 and the inactivation of the retinoblastoma (pRb) tumor suppressor protein, respectively [12]. In addition, as the expression of the oncogenes E6 and E7 from high-risk HPV is the primary cause of CC, it has also been demonstrated that their continuous expression kept cancer cells alive and resistant to apoptosis and senescence [13,14]. Therefore, these two proteins are considered crucial therapeutic targets to fight against papillomavirus-caused cancers [15].

Chronic inflammation has been established as a cofactor implicated in the development of HR-HPV-associated cancers. In particular, the excessive reactive oxygen species (ROS) and reactive nitrogen species (RNS) produced under chronic inflammatory status can induce DNA damage and genomic instability, favoring the viral genome integration and the initiation of the malignant phase [16]. 

Several vaccines have been developed and exist today on the market (bivalent, quadrivalent, or nonavalent) based on the HPV genotypes targeted and all have a similar mode of action: they all induce a specific humoral response, which impedes the uptake of HPV by the basal epithelial cells of the cervix [17]. Apart from vaccines as a prophylactic method, there is currently no available specific treatment to cure patients already infected by HR-HPV. Anti-inflammatory drugs, by targeting cyclooxygenase-2 (COX-2)/prostaglandin E2 (PGE2) signaling, can reduce HPV-related chronic inflammation, thus preventing HPV integration and reducing the risk of CC development [18,19]. Other strategies aimed at stimulating immune responses have been developed and are still under investigation, such as the topical agent imiquimod, which acts as a toll-like receptor 7/8 (TLR7/8) agonist [20]. Indeed, in addition to being a less invasive and cheaper option than many other treatment modalities, imiquimod has been shown to act as an immune response modifier agent through its capability to directly activate innate immune cells and induce the production of cytokines, such as interferon (IFN)-α, tumor necrosis factor (TNF)-α, interleukin (IL) -1α, IL-1β, IL-6 or IL-8, in peripheral blood mononuclear cells (PBMCs) [21,22]. Cytokines are immune mediators playing important roles in host defense against HPV infection by promoting clearance and preventing viral persistence but altered pro-inflammatory cytokine levels can predispose to a chronic inflammatory status that is prone to CC initiation and progression. Contradictory data exist about the cytokine profile of the genital tract microenvironment in HPV-positive patients, and the relationship between their concentrations and their roles in HPV clearance or persistence remains to be clarified [23].

There is ultimately a need to develop more suitable preclinical models to understand the complex mechanisms between host immunity and HPV clearance/persistence to identify the best therapeutic targets for each stage. In parallel, an accurate diagnostic, based on the evaluation of the clinical aspects of the lesion(s), biopsy results, and other biological analyses, is crucial to determine the stage and the extent of the disease and help in the choice of the appropriate therapeutic strategy. Currently, HPV-associated genital warts can either be managed by the intervention of a medical doctor, usually through cryotherapy, trichloracetic acid, or surgical removal, or by self-applied patients therapies, through topical-acting agents such as podophyllotoxin, imiquimod, or sinecatechin (polyphenon E) if the wart clusters are small [24]. Unfortunately, virus recurrence after these treatments is still 30-40% [25]. As it is now strongly supported that altered cell-mediated immunity is associated with increased HPV infection and disease [26], there is still a need for therapies that can: (i) support HPV clearance and (ii) minimize the risk of CC development. In this regard, micro-immunotherapy (MI), as an immune-modulatory-based approach, is an interesting option for the treatment of HPV-related infections.

The use of MI medicines (MIM) could be seen as an interesting treatment option in order to reduce the principal mediators of chronic inflammation. In vivo studies have already shown interesting systemic and local anti-inflammatory effects in MIM-treated mice without any signs of toxicity [27,28,29]. In addition, MIM display a sequential and multi-target strategy that allows simultaneous interventions on several signaling molecules and immune mechanisms involved in the etiopathogenesis of immune-mediated diseases. 

Micro-immunotherapy medicines consist of sugar pillules, also called globules, impregnated with ethanolic preparations consisting of low doses (LD) and ultra-low doses (ULD) of cytokines, immune mediators, and specific nucleic acids (SNA^®^—hereafter referred to as SNA). For patients’ convenience, the globules are packaged into capsules, intended to be opened and administered through oromucosal delivery in a fasted state. Another specificity of MIM relies on the sequential capsules’ administration; indeed, each MIM encompasses different globule-containing capsules, each one having to be taken every day, in a successive order (capsule-1 on day 1, capsule-2 on day 2, etc.). 

In the specific context of HPV infection, the MIM 2LPAPI^®^ (hereafter referred to as 2LPAPI) is prescribed in some countries as an adjuvant of immune regulation in cases of anogenital infections caused by HPV, diagnosed by a doctor, and confirmed by biology. The complete formulation of this medicine encompasses five capsules (hereafter named MIM-1, MIM-2, MIM-3, MIM-4, and MIM-5), all of them being made of the same starting material but at different Centesimal Hahnemannian dilutions (CH). The complete sequence of 2LPAPI is as follows (also see Table 1): human recombinant (hr)-IL-1β either employed at 10 or at 17 CH (10-17 CH); hr-IL-2 (10-17 CH), hr-IFN-α (10-17 CH), cyclosporin A (CsA) either at 7 CH, 10 CH, or 17 CH (7-10-17 CH), RNA extracted from plants (10-18 CH), SNA-HLA-II ([10-18 CH], an SNA intended to target human leukocyte antigen [HLA] II), and SNA-PAPI ([10-18 CH], an SNA intended to target several genotypes of HPV, including HPV 6, 11, 16 and 18). From a clinical standpoint, the efficacy of 2LPAPI has previously been evaluated in an observational study performed in a small group of HR-HPV-positive women aged from 20 to 45 years old. This follow-up showed that the treatment (one capsule per day for 6 months) was safe, improved the clearance, and allowed the regression of cervical lesions compared to the control group [30]. An ongoing double-blind clinical study was further set up to evaluate the efficacy of the medicine in the clearance of genital HR-HPV infections compared to placebo (NCT04232917). In parallel to the aforementioned studies, the current preclinical research study aimed to investigate the mode of action of this medicine, assessing its effects in two HR-HPV preclinical models. 

The present in vitro study is thus divided into three parts: (i) the first one is intended to develop an in vitro immunological model of HPV-16 to better understand the responses of the immune cells to viral proteins derived from the HPV 16-L1 capsid-protein (HPV16_(L1)_). Thus, human PBMCs were challenged with HPV16_(L1)_, and the lympho-proliferation, the cytokine secretion, and the expression of activation markers were assessed. (ii) The second part aimed to evaluate the immune-modulatory effects of the five capsules composing the sequential 2LPAPI MIM in our HPV16 _L1_-model. In the last part (iii), the effects of this medicine were finally assessed in HeLa cells, a CC-derived cell line. Multiple studies reported the HeLa cells as a valid research model for the study of HPV carcinogenesis, as well as for evaluating the potential benefits of therapies aiming at preventing CC development and progression [31,32,33]. Indeed, this cell line has multiple copies of viral HPV type 18 DNA integrated into the genome and can be referred to as HPV-18-positive cells [34].

## 2. Materials and Methods

### 2.1. Tested Items

The tested micro-immunotherapy medicine (MIM) 2LPAPI is a homeopathic medicinal product consisting of sucrose–lactose globules impregnated with ethanolic preparations of cytokines, cyclosporin A (CsA), plant-extracted RNA, and specific nucleic acids (SNAs). The complete formulation of this medicine encompasses 5 different capsules (here referred to as MIM-1, MIM-2, MIM-3, MIM-4 and MIM-5, respectively), intended to be taken in a sequential manner (MIM-1 on Day 1, MIM-2 on Day 2, etc.). All of the capsules are composed of the same starting material, but prepared at different LD and ULD; thus, each capsule has its own composition (described in Table 1). The 2LPAPI and the vehicle (Veh.) capsules were manufactured by Labo’Life España, Consell–Mallorca, Spain, as previously described [29,35], and have been provided for investigational purposes. The overall composition of 2LPAPI is as follows: human recombinant (hr)-interleukin (IL)-1β at 10 or 17 CH (10-17 CH), hr-IL-2 (10-17 CH), hr-interferon (IFN)-α (10-17 CH), CsA either at 7 CH, 10 CH, or 17 CH (7-10-17 CH), RNA extracted from plants (10-18 CH), SNA-HLA-II (10-18 CH), and SNA-PAPI (10-18 CH). Regarding the Veh. capsules used as controls, previous publications described how those are produced in order to provide a suitable control for preclinical research [27,29,35,36,37]. In the current study, MIM- or Veh.-capsules were freshly diluted in 100 mL of culture medium to reach the final sucrose-lactose concentration of 11 mM.

### 2.2. Assessment of the Immune-Modulatory Effects in Peripheral Blood Mononuclear Cells

Healthy volunteers were enrolled by the French Blood Bank Center (Etablissement Français du Sang, [EFS], Pays de Loire, Nantes, France, www.efs.sante.fr, accessed on 2 February 2024), a public institution under the responsibility of the French Ministry of Health, and informed consent was obtained from all individuals. According to French ethics laws, blood donation is based on voluntary participation, non-remuneration, anonymity and non-profitability. A declaration regarding the use of blood samples from blood volunteer donors for non-therapeutic research purposes was submitted to the French Ministry of Research, and the agreement number CPDL-PLER-2023 009 was assigned. Thus, all blood samples were approved by the EFS Blood Bank Center, with written informed consent obtained from all the donors, in accordance with the Declaration of Helsinki.

#### 2.2.1. Treatment with HPV16_(L1)_

On day 0 (D0), peripheral blood mononuclear cells (PBMCs) from 3 healthy women donors (20, 21 and 24 years old, referred to as #1, #2, and #3, respectively) were incubated at the density of 200,000 cells/well in 96-well plates, in complete RPMI medium (Panbiotech, Aidenbach, Germany, P04-17500) supplemented with HEPES buffer (10 mM, Lonza, Basel, Switzerland, 17-737E), non-essential amino acids (1X, Lonza, 13-114E), sodium pyruvate (1 mM, Lonza, 13-115E), L-glutamine (2 mM, Biowest, Nuaillé, France X0550-100), human decomplemented serum AB (2% *v*/*v*, SIGMA, H3667-100) in the presence of HPV16_(L1)_ (PepMix^TM^ HPV16 (L1), JPT Peptide Technologies GmbH, Berlin, Germany) at the final dilution of 1/400 *v*/*v*. On D5, the cells were harvested, saturated with FcBlock solution (BD, 564220), immune-stained with fluorescent anti-CD3 (BioLegend, San Diego, CA, USA, 300325), anti-CD4 (BioLegend, 300519), and anti-CD8 antibodies (BioLegend, 344722), fixed, and analyzed by flow cytometry on a BD FACS Canto II (BD Biosciences, Franklin Lakes, NJ, USA), configuration 4/2/2.

#### 2.2.2. Cell Treatment in the Presence of HPV16_(L1)_

On D0, PBMCs from 6 healthy women donors (from 25 to 35 years old) were incubated at the density of 200,000 cells/well in 96-well plates in Roswell Park Memorial Institute (RPMI) 1640 medium (Panbiotech, P04-17500), added with 2% human decomplemented serum, 1 mM non-essential amino acids, 1 mM pyruvate, 2 mM L-glutamine, 10 mM 4-(2-hydroxyethyl)-1-piperazineethanesulfonic acid (HEPES) buffer, in the presence of HPV16_(L1)_ peptide mix at the final dilution of 1/450 *v*/*v*. On D2, either the Veh., MIM-1; -2; -3; -4 or -5 were added to the medium and incubated for the next 72 h at the final sucrose–lactose concentration of 11 mM. Controls with either HPV16_(L1)_ or IL-2 (20 ng/mL) were also run in parallel. On D5, the cells were harvested, saturated with FcBlock solution (BD, 564220), immune-stained with fluorescent anti-CD3, anti-CD4, anti-CD8, anti-CD71, anti-CD95, anti-CD28 and anti-HLA-DR antibodies (all purchased from BioLegend), fixed, and analyzed by flow cytometry. The discrimination of the cell sub-populations is as follows: CD4^+^: CD3^high^; CD4^high^; CD8^low^; SSC^low^, CD8^+^: CD3^high^, CD4^low^, CD8^high^, SSC^low^, CD4^−^/CD8^−^: CD3^high^; CD4^low^; CD8^low^; SSC^low^. The supernatants (SNs) were retrieved and the cytokine levels of IFN-γ, IL-6, and interferon- γ inducible protein (IP-10) were assessed by enzyme-linked immunosorbent assay (ELISA).

### 2.3. Assessment of the Cellular Viability and the Cell Confluence in a Model of HeLa Cancer Cells

**Figure 1:**
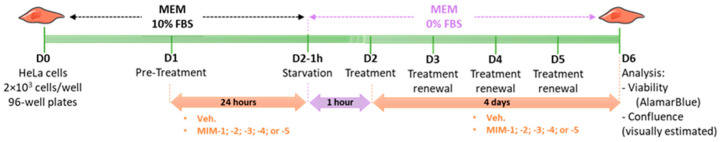
Representative scheme of the experimental protocol. Briefly, 2 × 10^3^ cells/well were seeded in a 96-well plate in minimum essential medium (MEM) containing 10% fetal bovine serum (FBS) on Day 0 (D0). The cells were then pre-treated for 24 h with either the vehicle (Veh.), micro-immunotherapy medicine (MIM)-1, -2, -3, -4 or -5- in 10% FBS, and starved for one hour in serum-free MEM on D2. After this step, the treatments were re-added to the medium and renewed every 24 h over the next 4 days. The cells were kept in a serum-free medium during the entire treatment duration. On D6, the cell viability was assessed by AlamarBlue reagent, and the confluence was visually estimated by microscopy. FBS: fetal bovine serum; MEM: minimum essential medium.

HeLa cells (human cervix epithelioid carcinoma) were supplied by the European Collection of Authenticated Cell Cultures (ECACC) (ref: #93021013, ECACC, Salisbury, UK) and cultured according to the supplier’s recommendations. Briefly, HeLa cells were cultured in minimum essential medium (MEM) (Gibco, ref: #11095-080, batch 2276790) supplemented with 10% fetal bovine serum (FBS) (Gibco, ref: #16000-044, batch 2208592RP) and maintained at 37 °C and 5% CO_2_. On D0, HeLa cells were seeded in a 96-well plate at a density of 2 × 10^3^ cells per well under normal conditions (10% FBS), which represents approximately 10% of cell confluency. This was a blind experiment. On D1, cells were pre-treated with diluted capsules of either the Veh., MIM-1; -2; -3; -4 or -5 under normal conditions (10% FBS) for 24h. On D2, cells were deprived in serum for 1 h (0% FBS). After 1h, the cells were treated with diluted capsules of either the Veh., MIM-1; -2; -3; -4 or -5, under starvation conditions (0% FBS). Treatments lasted four days and the medium was renewed every 24 h with fresh items. On D6, the cell viability was assessed with AlamarBlue cell viability reagent according to the supplier’s recommendations. Briefly, AlamarBlue reagent (Invitrogen, Waltham, MA, USA, ref: #DAL1025, batch 2284621) was directly added to the culture medium and incubated for 3 h at 37 °C in a cell culture incubator, protected from direct light. Results were recorded on a Synergy H2 plate reader (Agilent, Santa Clara, CA, USA), using the fluorescence excitation wavelength of 560 nm and an emission of 590 nm. The cell confluence was visually estimated by microscopy on a Nikon Eclipse TS100 (Nikon Corporation, Tokyo, Japan). Representative pictures of the wells were taken each day before treatment renewal for each condition using a 20× magnification. The complete protocol is illustrated in Figure 1.

### 2.4. Statistical Analysis

The graphs in the figures were performed with GraphPad Prism, Version 10.1.1.323 for Windows (GraphPad Software Inc., San Diego, CA, USA, updated on 29 November 2023). Authors have followed the recent recommendations of D.L. Vaux that encourage performing descriptive statistics instead of making statistical inferences when the number of independent values is small [38]. If the results of the in vitro studies are derived from only one, two, or three (*n* = 1, or *n* = 2, or *n* = 3) experiment(s), it is always better to include a full dataset, plotting data points and letting the readers interpret the data for themselves, rather than drawing statistical inferences, showing *p* values, standard error of the mean (S.E.M.), or results that are not representative. Thus, not all the data of this study were subjected to statistical analysis, and two-way ANOVA has solely been performed on PBMC analysis, as *n* = 6 donors were included in this part (see from Section 3.2, Section 3.3, Section 3.4, Section 3.5 and Section 3.6).

## 3. Results

### 3.1. HPV16_(L1)_ Stimulates the Lympho-Proliferation of Human Peripheral Blood Mononuclear Cells 

**Figure 2:**
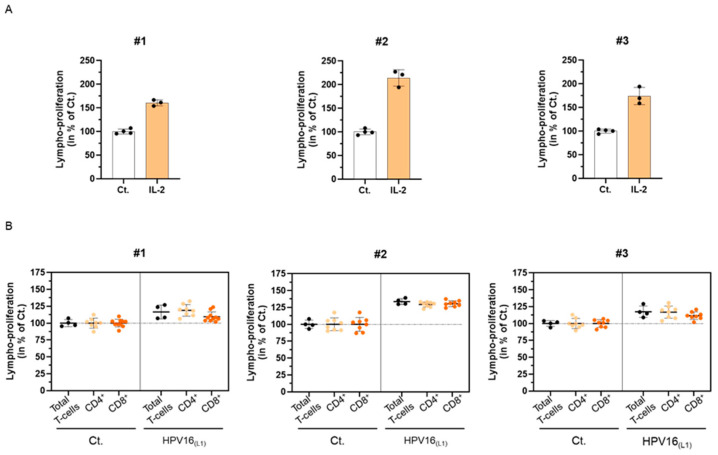
Influence of the L1-protein capsid of HPV 16 (HPV16_(L1)_) on the proliferation of human peripheral blood mononuclear cells (PBMCs) isolated from three women donors (**#1**–**#3**). (**A**) Lympho-proliferation in the untreated control condition (Ct.) and in the 20 ng/mL IL-2-treated condition for each of the assessed donors. Data are illustrated as the mean percentages ± standard deviation (S.D.) of the lympho-proliferation for *n* = 4 replicates for the Ct., and *n* = 3 replicates for the IL-2 conditions, obtained for each donor, the untreated Ct. condition being set as 100%. (**B**) Lympho-proliferation in the untreated control condition (Ct.) and in the HPV16_(L1)_ (1/400 *v*/*v*)-treated condition for each of the assessed donors. The proliferation of the total T-cells (black dots), CD4^+^ T-cells (light orange dots), and CD8^+^ T-cells (dark orange dots) was appraised. The data are presented as the mean percentage ± S.D. of the total cells counted after an incubation of 5 days; the Ct. condition being set at 100%. Each dot represents a technical replicate, and the dotted lines in (**B**) are drawn to highlight the effect of HPV16_(L1)_ in comparison with the untreated Ct.

In line with the ongoing clinical trial of 2LPAPI (NCT04232917), which enrolls women with high-risk (HR)-HPV infections, we wanted to assess the in vitro immune-related effects of this medicine on a suitable HPV infection model. Therefore, we collected blood samples of healthy donors to develop an immune-cell model of primary cells and set up a preliminary experiment, attempting to investigate the immunogenicity of a pool of 124 peptides derived from the L1-protein capsid of HPV 16 (HPV16_(L1)_). Thus, as a preliminary readout for our experiment, we assessed the proliferation of human peripheral blood mononuclear cells (PBMCs) exposed to HPV16_(L1)_. Briefly, PBMCs were retrieved from three women (#1, #2, and #3) and were either stimulated with 20 ng/mL IL-2, as a positive control for lympho-proliferation [39] or treated with HPV16_(L1)_. The cells were incubated for 5 days in those conditions before being harvested, fixed, immune-stained, and analyzed by flow cytometry. The proliferation of the total T-cells in the presence of IL-2 is illustrated in Figure 2A. As expected, such treatment induced a strong proliferation of the lymphocytes after 5 days of incubation in the three tested donors, with increments ranging from at least 60% and up to 114% depending on the donors (the untreated control condition being set at 100%). Figure 2B illustrates the lympho-proliferation in each of the donors for the total T-cells, as well as the ones of the CD4^+^ and CD8^+^ sub-populations, in HPV16_(L1)_ treatment-condition vs. control. With increases ranging from 17% to 34%, compared with the untreated baseline, HPV16_(L1)_ induced an overall milder lympho-proliferation than the one induced by IL-2 (Figure 2B, black dots). While this increase in lympho-proliferation was also observed in the two CD4^+^ and CD8^+^ sub-populations (Figure 2B, orange dots), it could be noticed, however, that the CD8^+^ cells displayed a weaker response than the CD4^+^ cells in the PBMCs retrieved from #3 and #1. Of note, the same treatments have been performed on PBMCs retrieved from *n* = 3 men donors, and the overall data collected from both sexes are illustrated in Supplementary Figure S1A,B. Interleukin-2 and HPV16_(L1)_ also increased the T-cell proliferation in the cells retrieved from men.

Altogether, these preliminary results allowed us to validate that the experimental protocol followed here with HPV16_(L1)_ was a good immune-stimulatory model for T-cell proliferation, thus allowing us to further assess the immune-modulatory effects of the capsules from 2LPAPI to investigate its possible mode of action.

### 3.2. HPV16_(L1)_ Stimulates the TNF-α Secretion in Human Peripheral Blood Mononuclear Cells 

**Figure 3:**
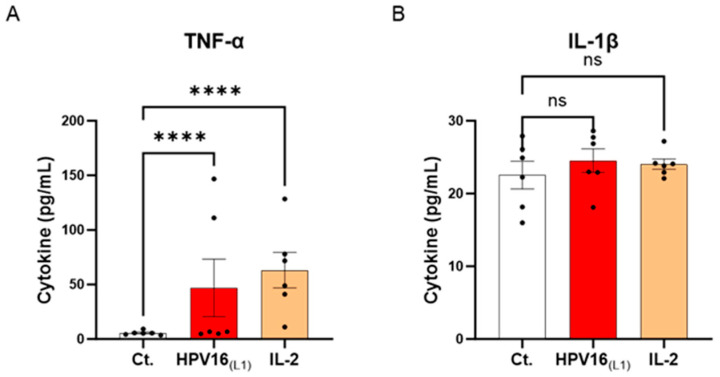
Effect of HPV16_(L1)_ and IL-2 treatments on the secretion of cytokines involved in the anti-viral response in human peripheral blood mononuclear cells (PBMCs). The secretion of (**A**) TNF-α and (**B**) IL-1β has been assessed by enzyme-linked immunosorbent assay (ELISA). The secretion levels of these cytokines have been assessed in the control condition (Ct.; white histograms) or after treatment with either HPV16_(L1)_ peptide pool (1/450 *v*/*v*; red histograms), or 20 ng/mL of IL-2 (orange histograms), within the supernatants (SNs) recovered from PBMCs isolated from women donors. Data are illustrated as the mean concentrations (in pg/mL) ± S.E.M. obtained for *n* = 6 donors in each treatment condition. Two-way ANOVA: **** *p* < 0.0001, ns: non-significant.

Cytokines are crucial immune mediators involved in inflammation. From one side, pro-inflammatory cytokines are necessary for viral clearance; however, in the context of HPV infection, these molecules are also the ones mainly responsible for a pernicious status of chronic inflammation, encouraging carcinogenesis and cervical carcinoma (CC) progression. Knowing this cytokine paradox, we wanted to investigate the effects of HPV16_(L1)_ on the cytokine profile of human PBMCs. Thus, the secretion levels of a panel of cytokines involved in inflammation and anti-viral immune responses (tumor necrosis factor (TNF)-α, interleukin (IL)-1β, IL-6, interferon-γ inducible protein (IP-10), interferon (IFN)-γ, IFN-α2, IL-12p70, and granulocyte macrophage colony-stimulating factor (GM-CSF)) were assessed. Thus, in this experimental setting, PBMCs from 6 women were retrieved and either left untreated, subjected to HPV16_(L1)_ peptide pool, or treated with 20 ng/mL of IL-2 for five days. At the end of the incubation period, supernatants (SNs) were recovered, and the secretion levels of the aforementioned cytokines were analyzed using an enzyme-linked immunosorbent assay (ELISA). The results are presented in Figure 3 and Supplementary Figure S2. Our results showed that, in PBMCs exposed to HPV16_(L1)_, there was an increase in the levels of TNF-α and IL-1β, two well-known primary pro-inflammatory cytokines, compared to the control (Figure 3A,B). In particular, TNF-α secretion increased in a statistically significant manner (Figure 3A).

These results suggest that our in vitro model of HPV-16-treated PBMCs mimics, at least partially, the pro-inflammatory conditions driving the low-grade inflammation related to persistent HR-HPV infections.

### 3.3. Actives from 2LPAPI Slightly Increased the Secretion of IL-6, IP-10 and IFN-γ in Human Peripheral Blood Mononuclear Cells Exposed to HPV16_(L1)_

**Figure 4:**
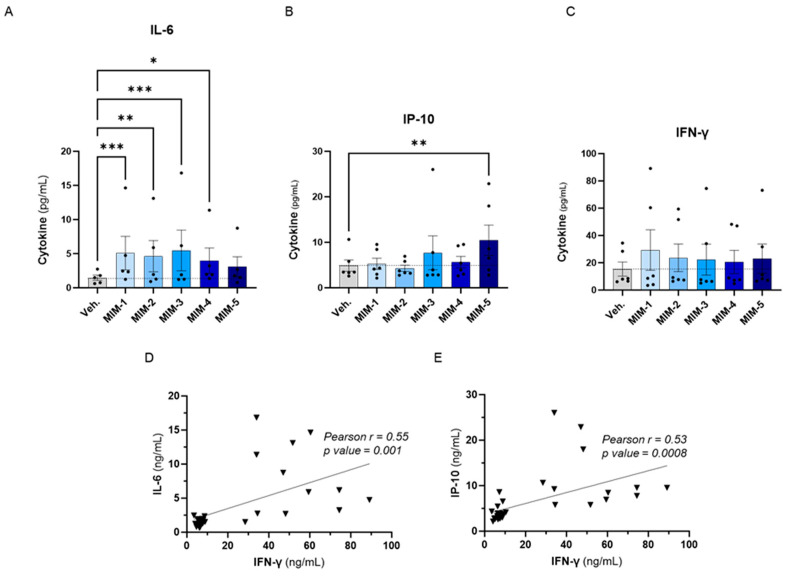
2LPAPI slightly increased the secretion of cytokines involved in the anti-viral response in a model of HPV16_(L1)_-treated peripheral blood mononuclear cells (PBMCs) from women. The secretion of: (**A**) IL-6, (**B**) IP-10, and (**C**) IFN-γ has been assessed by enzyme-linked immunosorbent assay (ELISA) after treatment with either the vehicle (Veh.), MIM-1; -2; -3; -4 or -5, in the presence of HPV16_(L1)_ (1/450 *v*/*v*), within the supernatants (SNs) recovered from PBMCs isolated from women. Data are illustrated as the mean concentrations (in pg/mL) ± S.E.M. obtained for n = 6 donors in each treatment condition. Two-way ANOVA: *** *p* < 0.001, ** *p* < 0.01, * *p* < 0.05. The dotted lines in (**A**–**C**) are drawn to highlight the effect of each treatment in comparison with the Veh. condition. (**D**,**E**) Pearson correlations between the cytokine concentrations retrieved in the SNs after treatment with the five 2LPAPI capsules. The correlation between IL-6 and IFN-γ (**D**) and between IP-10 and IFN-γ (**E**) are illustrated, and Pearson’s r scores and *p*-values are depicted in each graph.

To evaluate the effects of the five capsules of 2LPAPI on the cytokine secretion in our in vitro model of HPV16_(L1)_-treated PBMCs, we wanted to focus on cytokines involved in anti-viral immune responses; thus, the same panel as in Section 3.2. was assessed (IL-6, IP-10, IFN-γ, IL-1β, TNF-α, IFN-α2, IFN-γ1, IL-12p70, and GM-CSF). The effects of 2LPAPI on those cytokines are depicted in Figure 4 and Supplementary Figure S3, as the mean pg/mL ± standard error of the mean (S.E.M.) of the secretion obtained in each condition, for *n* = 6 donors. Interestingly, it can be observed that 4 out of the 5 tested capsules of 2LPAPI significantly increased the secretion of IL-6 (Figure 4A). The effects on IP-10 secretion have a different pattern: while MIM-1, MIM-2, and MIM-4 have barely affected IP-10 secretion, MIM-3 and MIM-5 have induced an increased secretion of this factor, even reaching statistical significance for MIM-5 (Figure 4B). All the capsules of 2LPAPI slightly increased the secretion of IFN-γ in a non-significant manner (Figure 4C). MIM-1 seemed to have the most IFN-γ-inducing effect, while the magnitude of the effects appeared quite similar for the 5 capsules as the means ranged from about 20 to 30 pg/mL in MIM-treated cells compared to the Veh.-treated (in which the mean is about 15 pg/mL). Of note, no major change in the secretion levels of IL-1β, TNF-α, IFN-α2, IFN-γ1, IL-12p70, and GM-CSF was noticed as a consequence of treatment with 2LPAPI capsules in the experimental conditions tested here. Interestingly, positive correlations between the levels of IL-6 and IFN-γ (r = 0.55, *p* = 0.001, Figure 4D), and between IP-10 and IFN-γ concentrations (r = 0.53, *p* = 0.0008, Figure 4E) were found amongst samples.

Overall, these results revealed that actives from the 2LPAPI can induce the stimulation of secretion of IL-6 and influence the levels of IFN-γ and IP-10 in the experimental conditions tested, even if the changes induced by the 2LPAPI capsules on IFN-γ did not reach statistical significance.

### 3.4. Actives from 2LPAPI Moderately Reduced the Proliferation of HPV16_(L1)_-Treated Human Peripheral Blood Mononuclear Cells 

**Figure 5:**
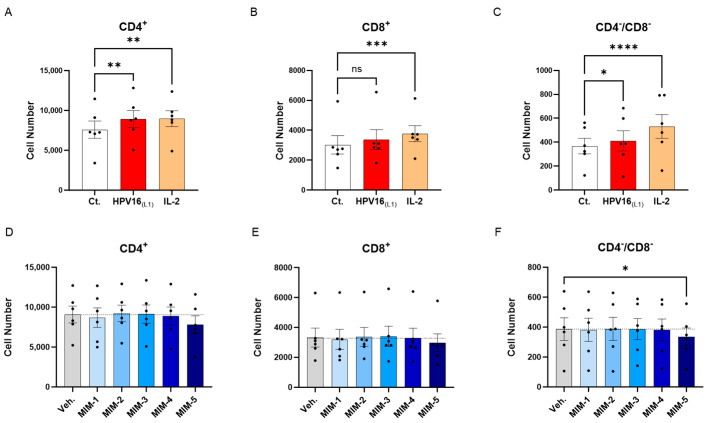
Effect of 2LPAPI on the proliferation of HPV16_(L1)_-treated peripheral blood mononuclear cells (PBMCs) from women. (**A**–**C**) Lympho-proliferation in the untreated control condition (Ct.; white histogram), in the HPV16_(L1)_ (1/450 [*v*/*v*])-treated condition (red histogram), and in the 20 ng/mL IL-2-stimulated condition (orange histogram), within the sub-populations of CD4^+^, CD8^+^, and CD4^−^/CD8^−^ T-cells, respectively. Data are illustrated as the mean cell number ± S.E.M. obtained for *n* = 6 donors in each treatment condition. (**D**–**F**) Lympho-proliferation after treatment with either the vehicle (Veh.), MIM-1; -2; -3; -4 or -5, in the presence of HPV16_(L1)_ (1/450 [*v*/*v*]), within the same sub-populations as in (**A**–**C**). Data are illustrated as the mean cell number ± S.E.M. obtained for *n* = 6 donors in each treatment condition. Each dot represents the mean value of the data collected from *n* = 2 replicate per donor. Two-way ANOVA: **** *p* < 0.0001, *** *p* < 0.001, ** *p* < 0.01, * *p* < 0.05, ns: non-significant. The dotted lines in (**D**–**F**) are drawn to highlight the effect of each treatment in comparison with the Veh. condition.

The previous experimental model was then used to directly appraise the effect of 2LPAPI on the proliferation of lymphocytes. Peripheral blood mononuclear cells from 6 women were thus left untreated (Ct.) or incubated for two days with (1/450 [*v*/*v*]) HPV16_(L1)_ alone, or HPV16_(L1)_ added with either the Veh., MIM-1, MIM-2, MIM-3, MIM-4, or MIM-5 for the next 72 h. The T-cell proliferation was evaluated by flow cytometry in the sub-populations of CD4^+^, CD8^+^, and CD4^−^/CD8^−^ T-cells. As mentioned in the previous section, a treatment with IL-2 alone was also employed as a positive control in this experiment. As illustrated in Figure 5A–C and Supplementary Figure S4, IL-2 treatment led to a statistically significant increase in the three analyzed T-cell subpopulations. Concerning the HPV16_(L1)_ treatment alone, it led to a similar statistically significant increase in the proliferation of those sub-populations, except in the CD8^+^ T-cells, in which the significance was not reached (Figure 5B). In the presence of HPV16_(L1)_, and after treatment with the 2LPAPI capsules, a consistent pattern was obtained in the three sub-populations of CD4^+^, CD8^+^, and CD4^−^/CD8^−^ T-cells, in which only MIM-5 seemed to decrease the proliferation (by 13.9% in the CD4^+^, 10.7% in the CD8^+^, and 13.3% in the CD4^−^/CD8^−^ T-cells; Figure 5D–F). In this experimental setting, this decrease in proliferation was only significant in the CD4^−^/CD8^−^ T-cells (Figure 5F). 

Overall, our first results seemed to show that in the context of HPV16_(L1)_ treatment, MIM-5 slightly reduced the proliferation of the CD4^+^, CD8^+^, and CD4^−^/CD8^−^ T-cells, thus possibly raising the question if this capsule could also influence the activation levels of these cells. To investigate this possible effect of 2LPAPI, we also assessed the expression of several surface activation markers under the same experimental conditions (see Section 3.5 and Section 3.6).

### 3.5. Actives from 2LPAPI Slightly Reduced the Expression of HLA-DR in HPV16_(L1)_-Treated Human Peripheral Blood Mononuclear Cells

**Figure 6:**
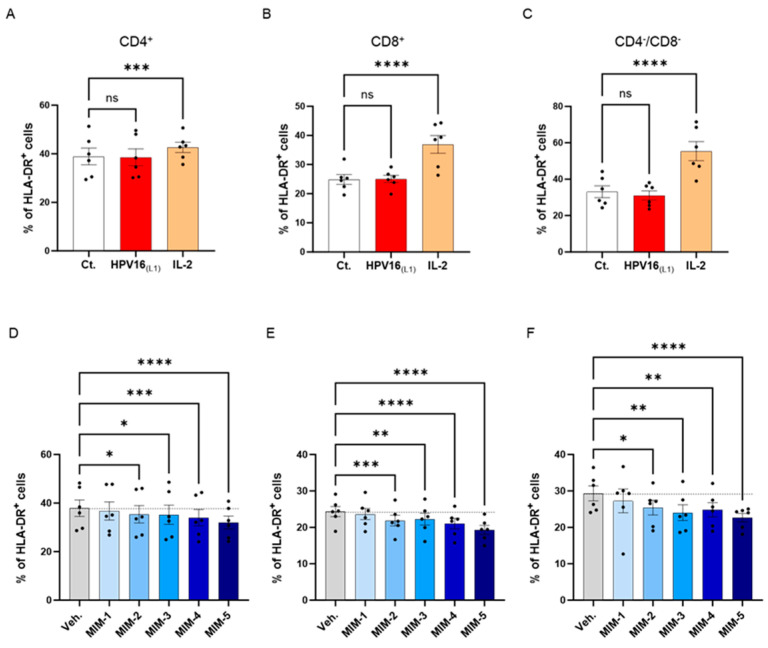
2LPAPI slightly reduced the expression level of HLA-DR in a model of HPV16_(L1)_-treated peripheral blood mononuclear cells (PBMCs) isolated from women. The expression of HLA-DR has been assessed in the untreated control condition (Ct.; white histogram), in HPV16_(L1)_ (1/450 [*v*/*v*])-treated condition (red histogram), and in 20 ng/mL IL-2-stimulated conditions (orange histogram), within the sub-populations of (**A**) CD4^+^, (**B**) CD8^+^, and (**C**) CD4^−^/CD8^−^ T-cells, respectively. The expression of HLA-DR has been assessed after treatment with either the vehicle (Veh.), MIM-1; -2; -3; -4 or -5, in the presence of HPV16_(L1)_ (1/450 [*v*/*v*]), within (**D**), the CD4^+^, (**E**), the CD8^+^, and (**F**), the CD4^−^/CD8^−^ T-cells sub-populations of PBMCs isolated from women. Data are illustrated as the mean percentage of positive cells ± S.E.M. obtained for *n* = 6 donors in each treatment condition. Two-way ANOVA: **** *p* < 0.0001, *** *p* < 0.001, ** *p* < 0.01, * *p* < 0.05, ns: non-significant. The dotted lines are drawn to highlight the effect of each treatment in comparison with the Veh. condition.

To investigate the possible effects of 2LPAPI on the activation status of HPV16_(L1)_-treated PBMCs, we assessed the expression of HLA-DR. It is worth noting that the 2LPAPI contains the specific nucleic acid (SNA)-HLA-II component at ultra-low dose (ULD), which has already been demonstrated to reduce the expression of HLA-DR in IFN-γ-stimulated HUVEC cells [40,41]. Therefore, evaluating the impact of 2LPAPI on HLA-DR expression in PBMCs treated with HPV16_(L1)_ peptide can provide valuable insights into its potential effectiveness in managing inflammation induced by HPV, as well as further validate its inhibitory effect on HLA-DR. Thus, in the same experimental setting as presented in Section 3.2, Section 3.3 and Section 3.4, the expression of HLA-DR was assessed by flow cytometry in the sub-populations of CD4^+^, CD8^+^, and CD4^−^/CD8^−^ T-cells. The results are presented in Figure 6, as the mean percentage ± S.E.M of positive cells for HLA-DR in each Ct., HPV16_(L1)_, IL-2-treatment condition and in the Veh.-/MIM-treatment condition. As illustrated in Figure 6A–C, while IL-2 induced a significant increase in the HLA-DR expression of the three T-cell sub-populations, HPV16_(L1)_ did not impact the expression of this marker. However, it appeared that in the three analyzed subsets of T-cells, capsules MIM-2 to MIM-5 significantly reduced the expression of this marker when compared with the Veh. (Figure 6D–F). Of note, MIM-5 seemed to be the capsule that induced the most powerful effect.

These data reinforce previous findings and confirm the hypothesis that MIM employing ULD of SNA-HLA-II in their compositions can reduce the levels of HLA-DR under different in vitro conditions. 

### 3.6. 2-LPAPI Could Reduce the Expression of Activation Markers of HPV16_(L1)_-Treated Human Peripheral Blood Mononuclear Cells 

**Figure 7:**
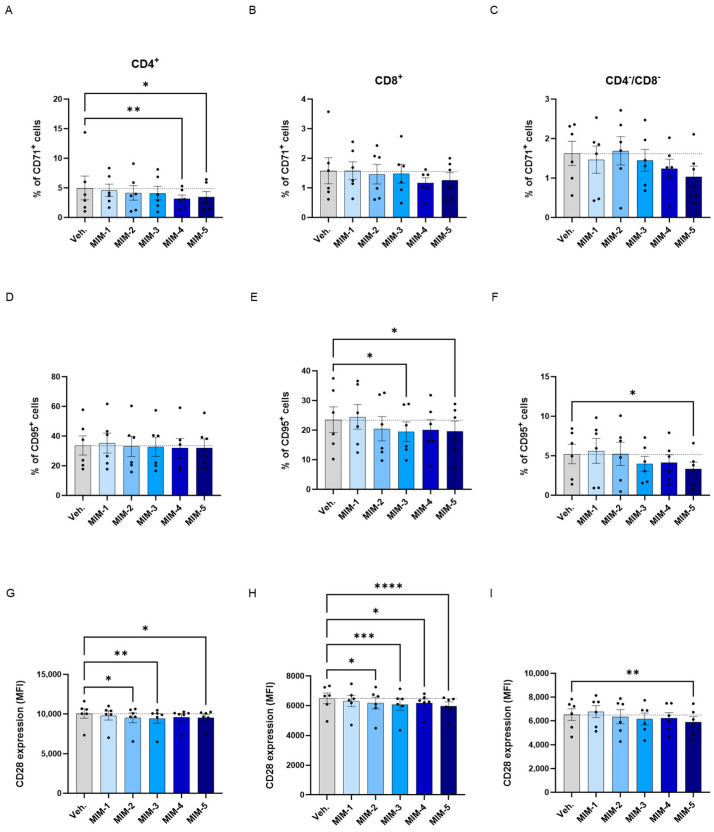
2LPAPI reduces the expression levels of membrane markers involved in T-cell activation in a model of HPV16_(L1)_-treated peripheral blood mononuclear cells (PBMCs) from women. The expression of CD71 (**A**–**C**), CD95 (**D**–**F**), and CD28 (**G**–**I**) has been assessed after treatment with either the vehicle (Veh.), MIM-1; -2; -3; -4 or -5, in the presence of HPV16_(L1)_ (1/450 [*v*/*v*]), within the CD4^+^ (**A**,**D**,**G**), CD8^+^ (**B**,**E**,**H**), and CD4^−^/CD8^−^ (**C**,**F**,**I**) T-cell sub-populations from PBMCs isolated from women. In (**A**–**F**), data related to the expression of CD71 and CD95 are illustrated as the mean percentage of positive cells ± S.E.M. obtained for *n* = 6 donors in each treatment condition. In (**G**–**I**), the results of the CD28 expression are expressed as the mean ± S.E.M. of the median fluorescence intensity (MFI) of the data collected from n = 6 donors in each treatment condition. Two-way ANOVA: **** *p* < 0.0001, *** *p* < 0.001, ** *p* < 0.01, * *p* < 0.05. The dotted lines are drawn to highlight the effect of each treatment in comparison with the Veh. condition.

In order to further investigate the effects of 2LPAPI in modulating the T-cell activation status, we complement the HLA-DR expression analysis by also assessing the expression of three other markers of T-cell activation. Thus, the levels of CD71, CD95 and CD28 were evaluated by flow cytometry in the same model of HPV16_(L1)_-treated sub-populations of CD4^+^, CD8^+^, and CD4^−^/CD8^−^ T-cells, as previously employed. The results are presented in Supplementary Figure S5 for the Ct., HPV16_(L1)_-, and IL-2-treatment conditions, and in Figure 7 for the Veh.-/MIM-treatment ones.

Overall, regarding the expression of CD71, it appeared that in the three analyzed subsets of T-cells, the tested capsules reduced the expression of this marker, except MIM-2, in the CD4^−^/CD8^−^ cells, when compared with the Veh. (Figure 7A–C). Of note, MIM-4 and MIM-5 seemed to be the capsules that induced the most powerful effect in reducing the percentage of CD4^+^/CD71^+^ cells, reaching statistical significance in this population.

Concerning the CD95, none of the tested capsules seemed to impact its expression within the CD4^+^ cells in comparison to the Veh. (Figure 7D), while MIM-2 to MIM-5 reduced its expression by about 15% within the CD8^+^ cells in comparison with the Veh. (Figure 7E). In addition, in the CD4^−^/CD8^−^ cells, MIM-3, MIM-4 and MIM-5 also showed a slight inhibitory effect on the expression of CD95 (Figure 7F).

Finally, the expression of CD28 has also been assessed in the same sub-populations, in which all the tested capsules reduced its expression in comparison with the Veh., with the exception of MIM-1 within the CD4^−^/CD8^−^ T-cells (Figure 7G–I). Altogether, these results seemed to show that, in an HPV16_(L1)_ treatment context, 2LPAPI, and particularly through MIM-5, could reduce the levels of CD71, CD95, and CD28 in several T-cell subsets (CD4^+^, CD8^+^, and CD4^−^/CD8^−^). These new data could possibly highlight an immune-modulatory effect of the medicine that downregulates the activation status of immune cells exposed to the HPV16_(L1)_ peptide pool.

### 3.7. Actives from 2LPAPI Could Lower the Proliferative Potential of HeLa Cells

The rationale behind the second part of this study relies on two main pieces of evidence: (i) serum deprivation is known to preferentially sensitize cancer cells to chemotherapeutic agents compared to near normal cells [42], and (ii), the posology of 2LPAPI requires a fasted state. Thus, we wanted to assess the effects of 2LPAPI on HeLa cell proliferation under serum starvation conditions. In order to optimize the setting of the experiments to further assess the effect of 2LPAPI on cancer cell viability, a pilot experiment was performed to evaluate the kinetics of cellular proliferation under different fetal bovine serum (FBS) concentrations. Briefly, HeLa (HPV 18-positive cells) were cultivated for 4 days, either in a serum-free medium or in the presence of 0.1%, 1%, or 5% (*v*/*v*) FBS. The cell confluence was monitored every 6 h during the whole duration of the experiment, thanks to the live content cell imaging technique, and the results are shown in Supplementary Figure S6. As expected, the different FBS concentrations influenced the proliferation of HeLa in a dose-dependent manner. The highest capacity of proliferation over time occurred when the cells were cultivated at 5% FBS (Supplementary Figure S6; dark green curves and histograms), and the lowest capacity of proliferation was observed in starvation conditions (without FBS) (Supplementary Figure S6; black curves and histograms). Importantly, these data highlighted the fact that HeLa cells were resistant to a so-called 4 day “long term” serum starvation, as they were still able to double their number after 4 days in culture under serum-free conditions.

In light of these results, we concluded that, even in a 4 day, “long-duration” condition, the complete serum starvation was still an experimental environment allowing viability studies, which represented a suitable model to test the effect of 2LPAPI in vitro. Thus, the treatment with either the Veh., MIM-1, MIM-2, MIM-3, MIM-4, or MIM-5 was then evaluated in serum-free media for 4 days. The details of the experimental conditions and the experimental scheme are depicted in Section 2.3 (see Figure 1). It is important to mention that this was a blind experiment. Briefly, HeLa cells were seeded at a density of 2 × 10^3^ cells/well on Day 0 (D0) and cultivated under normal culture conditions (10% FBS). On D1, a 24 h pre-treatment with either the Veh. or the tested capsules of 2LPAPI was performed in 10% FBS before the removal of both treatments and FBS for one hour as a serum deprivation step. The treatments were then added to serum-free media and were incubated with the cells for 4 days. The percentage of confluence was appraised every day via microscopy to obtain information about the kinetic of proliferation, and HeLa’s viability/proliferation was assessed with the well-known, non-toxic alternative to the commonly used MTT cell viability assay, AlamarBlue reagent, at the end of the incubation time [43].

**Figure 8:**
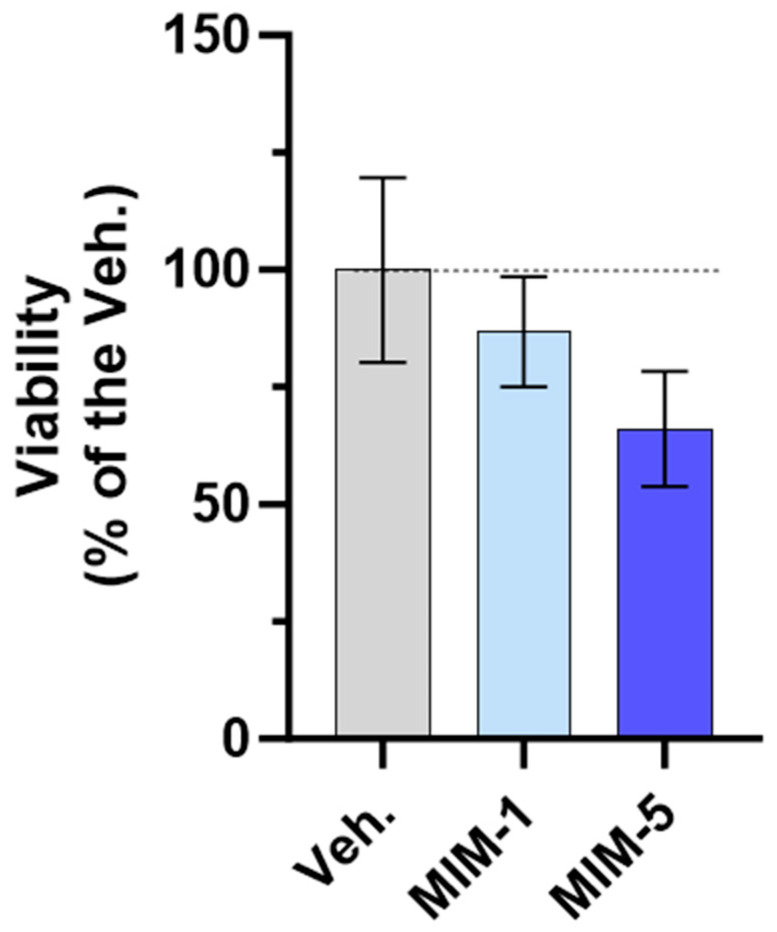
MIM-1 and MIM-5 lowered the cellular viability/proliferative potential in a model of starved HPV-positive cervical cancer cells. Cell viability/proliferative potential of HeLa cells was assessed via AlamarBlue reagent under the experimental conditions presented in Section 2.3 (see Figure 1) when treated with the vehicle (Veh.), MIM-1 or MIM-5. The results are presented as the mean percentages ± standard deviation (S.D.) of *n* = 6 replicates per condition, with the Veh. being set at 100%.

As shown in Figure 8, both MIM-1 and MIM-5 slightly decreased the cell viability compared with the Veh. (with a magnitude of about 15% and 35% for MIM-1 and MIM-5, respectively). Moreover, these results were confirmed by cell confluence observation. Indeed, the visual estimation of the cell confluence appeared reduced, at each time point, from D2 to D6 in the MIM-1-treated cells in comparison to the Veh. (Supplementary Figure S7A). Concerning MIM-5, the same downward trend was observed, from D3 to D6 (Supplementary Figure S7B). Of note, such growth inhibitory effect was not found with MIM-2, MIM-3, and MIM-4-treated cells, on D6 (Supplementary Figure S7D,E). In addition, the kinetics of proliferation suggested that maybe MIM-4 could have an effect on lowering the proliferative capacity from D2 to D5, while MIM-2 and MIM-3 showed no clear effect (Supplementary Figure S7D,E).

Altogether, these results finally suggest that, in the starvation condition tested here, MIM-1 and MIM-5 both display an ability to reduce the viability/proliferative potential of HPV-positive HeLa cells, as attested through a quantitative evaluation of the cell viability and confirmed by a visual evaluation of the cell confluence, all throughout the experiment, every single day.

## 4. Discussion

The present study sought to investigate the possible mode of action and the biological effects of the sequential micro-immunotherapy medicine (MIM) 2LPAPI, a therapeutic option that, according to a previous observational study, could be a valuable medicine for treating high-risk (HR)-HPV cervical lesions in women aged 25 years and above [30]. Considering the limited size of the follow-up and the lack of a placebo control group in the study, a double-blind versus placebo clinical study has been set up to comprehensively assess the efficacy and safety of 2LPAPI and is currently still ongoing. In this preclinical research study, the main objectives were to assess the effects of the actives composing the formulation of the MIM 2LPAPI in two HR-HPV-related models in vitro. The first model was obtained by exposing peripheral blood mononuclear cells (PBMCs) to a pool of peptides derived from the HPV 16 capsid (HPV16_(L1)_) for 72 h. To our knowledge, at the time this article was written, the aforementioned in vitro model of total PBMCs treated with HPV-16 peptides had not previously been described. Thus, the first goal here was to develop a proper HR-HPV model that could mimic the specific low-grade inflammation associated with persistent HPV infections. On the other hand, the second model employed here, the HeLa cancer cell line, has been widely used in fundamental research studies related to cervical cancer. Indeed, HeLa cells were originally collected from a woman who died from cervical carcinoma (CC) in 1951 [44], made up the first cell line established for fundamental and medical research, related or not to HPV, and also used during the preclinical development of HPV vaccines. Having HPV type 18 DNA integrated into their genome, those cells also represent one of the best cellular models to assess the potential effects of drugs in preventing HPV-induced carcinogenesis and/or treating HPV-induced cancer. Thus, HeLa cells were considered as a suitable model to investigate the in vitro effects of 2LPAPI.

As followed through the manuscript structure, our study had three main objectives: (i) to develop an in vitro HPV-16-immunological model to better understand the responses of the immune cells to HPV proteins; (ii) to investigate the effect of 2LPAPI from an immune standpoint, using the same in vitro model of HPV-16; (iii) to study the effect of the MIM on HeLa cell viability/proliferative capacity.

In the first part of the current study, we reported the immunological effects of HPV16_(L1)_ on PBMCs isolated from healthy women donors. Our investigations revealed that a 5-day incubation with HPV16_(L1)_ led to an increased lympho-proliferation in comparison with the untreated control (Figure 2B). In addition, we found that PBMCs exposed to HPV16_(L1)_ responded by increasing the secreted levels of TNF-α and IL-1β, even if it was statistically significant solely for TNF-α (Figure 3). Both cytokines mainly exert pro-inflammatory/immunostimulatory responses, and their role in HPV infection, carcinogenesis, and HR-HPV-associated CC is not yet fully elucidated. Vitkauskaite et al. reported significant increases in the circulating levels of several cytokines, including TNF-α and IL-1β, in patients with CC in comparison with healthy controls [45]. Indeed, these two cytokines, with their ability to recruit neutrophils, are associated with an increase in reactive oxygen species (ROS) production, which can drive carcinogenesis [46]. While more studies are necessary to validate this hypothesis, the oxidative stress produced in excess under chronic inflammation can facilitate the integration of the viral genome into the host genome and, ultimately, initiate the carcinogenic process [16].

With these findings, we pursued our research to appraise the immune-modulatory effect of 2LPAPI, in the presence of HPV16_(L1)_, with the rationale that this peptide pool could be a good model of a low-grade chronic inflammation. The exact mechanisms that are involved in the immunogenicity of HPV16_(L1)_ were out of the scope of this study, in addition to the delineation of a putative assembly of these peptides, but interestingly, concerning the L1 structure, it is worth mentioning that, by having expressed the L1 major capsid protein of HPV-16 in insect cells in vitro, Kirnbauer et al. were able to demonstrate that this protein self-assembles into 50 nm-virus-like particles, which are morphological analogs to native virions, in the absence of L2 or other papillomavirus proteins [47]. The effect of the five capsules composing the MIM on the cytokine secretion in our HPV-16 model is depicted in Figure 4, and interestingly, capsules MIM-1 to MIM-4 significantly increased the secretion of IL-6 (Figure 4A). On the other hand, MIM-5 was the only one that significantly increased the levels of interferon- γ inducible protein (IP-10), while the other capsules only slightly seemed to affect its secretion, without reaching statistical significance (Figure 4B). Similarly, the results of the effects of the tested capsules of 2LPAPI on IFN-γ showed that the treatment might slightly increase its levels but in a non-significant manner (Figure 4C). Despite the inter-individual variability usually observed in experiments using donor-derived primary cells, it is interesting to note that positive correlations were found among the samples between (i) the levels of IL-6 and IFN-γ (r = 0.55, *p* = 0.001, Figure 4D) and (ii) between the concentrations of IP-10 and IFN-γ (r = 0.53, *p* = 0.0008, Figure 4E). Overall, these results revealed that all the donors responded to the simultaneous exposure to HPV16_(L1)_ and 2LPAPI with a small/slight stimulation of the secretion of IL-6, IFN-γ, and, at least for one capsule, IP-10. Transposing these preclinical in vitro data in the context of HPV infection, this immunostimulant effect on cytokine secretion may be beneficial, as the three aforementioned cytokines could work synergically in promoting viral clearance without resulting in a status of chronic inflammation. This hypothesis should be supported by further clinical and fundamental research on the role of these cytokines in the context of HR-HPV. In sustaining our hypothesis, it is noteworthy to highlight that the treatment did not increase the levels of TNF-α and IL-1β (Supplementary Figure S3). This implies that the aforementioned putative markers of chronic inflammation were not affected under these experimental conditions and, thus, finally suggesting that the MIM modulates the host immunity in HPV-16 infections by slightly stimulating cytokines involved in anti-viral responses such as IL-6 [23]. While the increase is of about a few pg/mL, it is important to mention that immune cells can respond to those small concentrations of IL-6 [48], which correspond to the physiological circulating levels in healthy individuals [49], and the slight up-regulation of IL-6 in the context of HPV infection might be seen as a favorable effect that can sustain and promote the viral clearance. In addition, the positive correlation found between (i) the secretion of IFN-γ and IL-6 (Figure 4D) and (ii) the secretion of IFN-γ and IP-10 (Figure 4E) drew our attention, as IFN-γ has been demonstrated to upregulate IL-6 production via the Janus kinase 1/2 (JAK1/2)—signal transducer and activator of transcription 1 (STAT1) pathway in human innate immune cells [50]. Extrapolating these results within the local environment of the cervix, it is finally worth mentioning that a study conducted at the University of Hawaii found an association between increased cervical levels of TNF and other immunological markers, such as IL-10, IL-12, and macrophage inflammatory protein-1 α (MIP-1α), and a reduced likelihood of HPV clearance [51]. Even if, in the current work, our immune model derived from PBMCs is not directly transposable to the local cervical microenvironment, our preclinical data can, at least, bring about new knowledge explaining how 2LPAPI could modulate the secretion of some markers involved in chronic inflammation and anti-viral responses, opening novel perspectives for future research. In addition, to corroborate our hypothesis on the mode of action of 2LPAPI, an interesting study reported significantly higher levels of IP-10 in the cervicovaginal secretion of women that efficiently eliminated the virus compared to those that remained HPV-positive at the end of the study, suggesting a potential role of IP-10 in the HPV clearance [52]. 

Concerning our results about IFN-γ, and even if the trends are quite small, such an increase is still promising regarding the crucial role played by IFN-γ in the clearance of HPV. Upon infection with HPV, the immune system responds by releasing IFN-γ, which activates immune cells and initiates an anti-viral response by stimulating the production of anti-viral proteins, such as the so-called interferon-induced transmembrane proteins (IFITMs), tripartite motif (TRIM)5α, MXs or apolipoprotein B mRNA-editing enzyme catalytic polypeptide-like 3 (APOBEC3), that restrict the entry, endosomal fusion, viral uncoating, and nucleocapsid transport, inhibit viral replication and disrupt viral pathogenesis [53]. Additionally, IFN-γ enhances the activity of natural killer (NK) cells and cytotoxic T-cells, which are essential for eliminating HPV-infected cells, and promote the efficient clearance of the virus. It is, thus, interesting to pinpoint that 10 ng/mL IFN-γ was demonstrated to efficiently prevent HPV-16 pseudovirus infection of HaCaT cells, a spontaneously transformed human keratinocyte cell line [54]. 

In an attempt to pursue our investigations and evaluate the immune-modulatory effect of 2LPAPI, we wanted to focus our attention on the adaptive immune cells sub-populations. Thus, we appraised the ability of 2LPAPI actives to modulate the proliferation of three subsets of T-cells: the CD4^+^, the CD8^+,^ and the CD4^−^/CD8^−^. As this last subset of double-negative T-cells plays important roles either in protecting or in promoting inflammation and virus infection, in addition to a potential for malignant proliferation [55], we thought that its inclusion in the analysis panel would be important. We thus showed that, amongst all the analyzed T-cell sub-populations, MIM-5 was the only one able to reduce the proliferation, especially within the CD4^−^/CD8^−^ cells, in which its effect reached statistical significance (Figure 5D–F). Regarding this result, we could hypothesize that the presence of IFN-α at 17 CH may have contributed, at least partially, to the immune-modulatory effect of this capsule on T-cells. Indeed, it has been reported that IFN-α injection prolonged the proliferation and the expansion of Ag-specific CD8^+^ T-cells in an ovalbumin-immunized mice model [56]. According to the previously published data on ultra-low-dose (ULD)-based MIMs [27,36,37], we can hypothesize that IFN-α (17 CH), which, out of the five assessed capsules here, is exclusively employed in MIM-5, could have reduced the IFN-α expression and/or its biological activity, thus lowering the T-cell proliferation. 

From the results presented in Figure 6, we showed that 2LPAPI slightly inhibited the expression of the T-cell activation marker human leukocyte antigen (HLA)-DR. For instance, while the expression of HLA-DR was not increased by HPV16_(L1)_ alone (Figure 6A–C), MIM-2, -3, 4-, and -5 all significantly reduced its expression in the three assessed sub-populations of T-cells (Figure 6D–F). Of note, 2LPAPI contains specific nucleic acid (SNA)-HLA-II, either at 10 CH or at 18 CH, in conjunction with other actives. Interestingly, the ULD of the SNA-HLA-II, as used in MIM, have been reported to reduce the expression of both HLA-DR and HLA-DP in IFN-γ-stimulated endothelial cells and lipopolysaccharide (LPS)-treated macrophages, respectively [40,41]. Thus, the presence of this SNA at ULD in those capsules may also explain the current results, i.e., a similar reduction in HLA-II expression in our MIM-treated-HPV16_(L1)_-stimulated PBMCs. In addition to HLA-DR, a consistent pattern of reduction in the expression of CD71 was found in all the three T-cell sub-populations analyzed, CD4^+^, CD8^+^, and CD4^−^/CD8^−^, after treatment with MIM-4 and MIM-5 (Figure 7A,D,G). Moreover, the expression of the CD95 marker was reduced in CD8^+^ and in CD4^−^/CD8^−^ T-cells in a statistically significant manner in the MIM-5-treatment condition when compared with the Veh. (Figure 7E,F). Finally, MIM-2, -3, 4-, and -5 all significantly reduced the CD28 expression in the three assessed T-cells to different extents depending on the considered sub-populations (Figure 7C,F,I). Overall, and in accordance with the results of lympho-proliferation (Figure 7D–F), it can be observed that MIM-5 was the one that reduced the expression of the four activation markers assessed, HLA-DR, CD71, CD95, and CD28, the most consistently within the three T-cell subsets of interest. The decreased expression of HLA-DR, CD71, CD95, and CD28 on T-cells suggests that these cells may be functionally less activated by the specific HPV-16 peptide stimuli. 

While more investigations are needed, with these findings, we can hypothesize that 2LPAPI may work as an immune adjuvant to promote the clearance, and at the same time, it may contribute to lessening the risk of CC carcinogenesis by lowering the activation state and the risk of the initiation of the chronic inflammation state induced by HR-HPV infections. 

The HR-HPV-associated CC risk is well-documented by virological, molecular, clinical, and epidemiological studies [57]. Thus, in the last part of the study, we wanted to appraise the in vitro effect of 2LPAPI in a model of CC cells in which the carcinogenic process was initiated by HPV. Specifically, for our chosen model, HeLa are HPV-18-positive cells containing the viral genome integrated into the host DNA; thus, any effect of 2LPAPI on cell growth and/or on cell viability in such model could potentially attest to an effect on the expression of viral genes. Amongst the ingredients of 2LPAPI, we can primarily focus our attention on the SNA-PAPI as this particular SNA is supposed to target DNA sequences of different types of HPV, including HPV-18.

On the other hand, from a clinical perspective, fasting has often been associated with conventional cancer treatment, as tumor-starvation therapies are quite promising and give encouraging clinical results, especially within the context of gynecological cancers [58]. Indeed, beneficial effects on the quality-of-life scores have been reported when this method was associated with chemotherapy in gynecologic malignancies [59]. Transposed to preclinical settings, the effect of serum or food starvation in sensitizing cancer cells has been widely demonstrated, respectively, in vitro and in vivo [60,61,62]. However, recent studies reported that CC cells are still quite resistant to serum starvation [63], making it difficult to obtain efficient therapies for CC management, even under environmental stress conditions such as food starvation. Interestingly, it has also been shown that HPV infection exacerbates the negative effects of starvation on the development of *Chlamydia trachomatis* [64], another important risk factor for CC initiation [65]. Altogether, this body of literature supports the fact that the serum depletion state further used in the current study is coherent regarding the pathological context of the cellular model used here. In addition, as MIM administration is best suited in a fasted state, the serum-free culture conditions used here might also have mimicked the ideal administration mode for 2LPAPI, as analogies can be made between the cellular pathways involved in food deprivation and cellular starvation in the context of cancer [66]. Nonetheless, in our model of serum-starved HeLa cells, both MIM-1 and MIM-5 reduced the cell viability and the cell confluence after 5 days of treatment in comparison with the Veh. (Figure 8). MIM-1 might have worked thanks to its specific employment of SNA-PAPI (18 CH), which is supposed to target key sequences of HPV to lower their expression. Moreover, it is also possible that the presence of ULD of IFN-α within the MIM formulation could also have played a role in the inhibitory effect of 2LPAPI on cell viability and cell confluence. It has previously been reported that IFN-α is able to inhibit the proliferation of HPV 16-immortalized human keratinocytes [67]. Another study conducted on HeLa cells also suggested that 1 × 10^3^ IU/mL IFN-α selectively inhibited cytoplasmic HPV 18 mRNA [68]. These results were confirmed by Perea et al., who even suggested that, as IFN-α did not affect HPV 18 transcripts stability nor did mediate its inhibitory effects through HPV 18 enhancer sequences, it could, however, possibly repress nascent viral transcripts through regulatory cellular flanking regions [69]. Nonetheless, our pilot data can open the way to further investigations on this HPV-specific active substance of 2LPAPI. The role of IFN-α and its use at ULD in 2LPAPI has yet to be elucidated; nonetheless, our pilot data can open the way to further investigations on this HPV-specific active substance of 2LPAPI. 

The relationship between HPV and the immune system is still a growing research area, but it is now established that HPV infection has repercussions on cells from both innate and adaptive immunity. Indeed, this virus largely impacts immune-related processes such as macrophage differentiation, activation and maturation of dendritic cells, T-cell functions, the balance between type 1 T-helper cells (Th1) and Th2 cells, or even regulatory T-cell (Treg) infiltration [70]. Our in vitro PBMCs-derived immunological model of HPV-16 brings additional knowledge on how the virus could influence immune cell functions, creating novel perspectives for further research. While peripheral blood studies do not accurately represent the immune markers present at the specific site of HPV infection [71], it is interesting to have discovered this pattern of secreted cytokines induced by the HPV-16 peptide mix, especially the TNF-α and IL-1β upregulation that may explain, at least partially, one of the mechanisms involved in the chronic inflammation induced by HR-HPV. 

The major body of our study was dedicated to understanding the mode of action of 2LPAPI, an MIM authorized in Belgium and Luxemburg as an adjuvant to sustain the immune system in cases of anogenital infections caused by HPV, diagnosed by a doctor, and confirmed by biology. The results of this study point out that the medicine might promote viral clearance by slightly stimulating the secretion of IL-6, IP-10, and IFN-γ and contribute to impede the initiation or reduce the chronic inflammatory status associated with CC carcinogenesis.

While the model used in this research has limitations, as typically, peripheral blood studies do not accurately represent the immune markers present at the specific site of HPV infection, the current results provided new insights into the in vitro effect of 2LPAPI on PBMCs retrieved from women [71]. It is, thus, crucial to conduct complementary studies in the future, which will directly assess the effect of 2LPAPI in the tissue affected by the infection, considering the localized nature of HPV. In addition, and as highlighted by Trimble et al., it is also important to keep in mind that during the course of HPV infection, HPV antigens, which are necessary for both the initiation and the persistence of the disease, are not presented systemically in a robust manner and that clinically relevant immune responses are not always reflected in the peripheral blood [71]. While the systematic review and meta-analysis from Litwin et al. concluded that the infiltration of T-cells in HPV-infected and pre-cancerous epithelium might decrease due to effective immune evasion [72], the authors also mentioned that their analysis is unable to explain the factors behind the varying adaptive immune responses to HPV infection among individuals and that these factors likely include both environmental aspects like co-infection with other sexually transmitted infections as well as genetic factors such as HLA. On that note, and from the opposite side of the spectrum, in a chronic inflammatory context, considered as one of the driving factors for carcinogenesis, maintaining the expression of the HLA-DR receptor could also be seen as a valuable asset, as it could favor the mediation of the anti-tumor immunity. Indeed, as reported by Höhn et al., CD4^+^ tumor-infiltrating lymphocytes in CC have the ability to recognize E7 peptides provided by HPV in an HLA-DR-restricted background [73]. Nonetheless, several connections and interpretations of our results could be made in an attempt to explain the relationship between the immune cells and the CC cells, pinpointing the complementarity of the two models employed here. For instance, one interesting finding that could resonate with the two models that we have studied here is that a subgroup of CD71^+^ was discovered within CC cells that showed a higher presence of the HPV-E6 protein [74]. Importantly, the increased presence of the CD71^+^ population was linked to heightened tumorigenic characteristics. Keeping this in mind, it could be valuable, in the context of HPV, to consider CD71 as a good target to inhibit the aggressiveness of CC. Even if our results reported an ability of 2LPAPI and especially MIM-4 and -5 to reduce the expression of this receptor within T-cell sub-populations (Figure 7A–C), it could further be hypothesized that a similar effect of our medicine could be mimicked in cancer cells, opening a new perspective for further research. Moreover, in the context of genital warts and pre-cancerous cervical lesions, HLA-DR targeting could be an effective strategy, as its expression appears to be increased through the carcinogenic process of cervical cancer [75]. For instance, transcriptomics profiles and protein expressions were assessed in samples from normal cervixes, HPV types 16/18-positive, low-grade CIN (LGCIN), high-grade CIN (HGCIN), and squamous cell carcinoma (SCC), demonstrating upregulations of CD74 and HLA-DRA from normal cervix to CIN with the highest in SCC [76]. 

## 5. Conclusions

In this first in vitro study about the micro-immunotherapy medicine (MIM) 2LPAPI, the five capsules composing the medicine (referred to as MIM-1, -2, -3, -4 and -5) were tested, and to understand their possible mode of action, a double axis of research was followed. At first, we aimed to evaluate the immune-modulatory effect of those MIM capsules in a model of human peripheral blood mononuclear cells (PBMCs) derived from the blood of women donors that were challenged with an HPV16 peptide mix, HPV16_(L1)_). In the second part, our principal objective was to evaluate the effects of the medicine on high-risk (HR)-HPV-positive cells in which the virus is integrated into the genome. Thus, we assess the efficacy of this medicine in reducing the viability of the HPV-18-positive cervical carcinoma (CC)-derived HeLa cells. 

From an immune standpoint, our results showed that the 2LPAPI medicine slightly increased the secretion of IL-6, IFN-γ, and IP-10—the three cytokines involved in the host immune responses against viral infections—in HPV16_(L1)_-treated PBMCs. These results were accompanied by the modulatory effect of the 2LPAPI on T-cells retrieved from the same model. While the unique MIM-5 capsule reduced the proliferation of T-cells, MIM-1; -2; -3; -4, and -5 all displayed an overall ability to diminish the expression of the activation markers CD71, CD95, HLA-DR, and CD28, possibly suggesting an inhibitory effect counteracting the HPV16_(L1)_-induced immune-activation. 

Put into the context of HR-HPV infections, the duality of the immune-modulatory effect of 2LPAPI is interesting because, by (i) lowering the expression levels of the activation markers of HPV-challenged immune cells, the medicine could contribute to preventing a cancer-prone chronic inflammatory state, and at the same time, (ii) by slightly increasing the secretion of anti-viral cytokines, 2LPAPI could also promote viral clearance. Finally, our investigations in CC-derived cells reported the effect of MIM-1 and -5, in reducing the viability of serum-starved HeLa cells after four days of treatment. 

Taken together, these results shed light on the possible mode of action of 2LPAPI in the framework of HPV infections. 

## Figures and Tables

**Table 1:** Composition of the capsules from the 2LPAPI sequence.

Starting Material (CH)	MIM-1	MIM-2	MIM-3	MIM-4	MIM-5
hr-IL-1β	10	10	17	10	10
hr-IL-2	10	10	10	17	10
hr-IFN-α	10	10	10	10	17
RNA	10	18	10	10	10
CsA	10	10	7	17	10
SNA-PAPI	18	10	10	10	10
SNA-HLA-II	10	10	10	10	18

CH: centesimal Hahnemannian dilution; CsA: cyclosporin A; HLA: human leukocytes antigen; hr: human recombinant; IFN: interferon; IL: interleukin; MIM: micro-immunotherapy medicine; RNA: ribonucleic acid; SNA: specific nucleic acid.

## Data Availability

The data of the current study are available from the corresponding author upon reasonable request.

## Supplementary Materials

The following supporting information can be downloaded at: https://www.mdpi.com/article/10.3390/cancers16071421/s1. Supplementary Figure S1: Influence of the L1-protein capsid of HPV 16 (HPV16_(L1)_) on the proliferation of human peripheral blood mononuclear cells (PBMCs) isolated from (A) men and (B) women donors; Supplementary Figure S2: Effect of HPV16_(L1)_ and IL-2 treatments on the secretion of cytokines involved in the anti-viral response in peripheral blood mononuclear cells (PBMCs) retrieved from women; Supplementary Figure S3: Effect of 2LPAPI on the secretion of cytokines involved in the anti-viral response in a model of HPV16_(L1)_-treated peripheral blood mononuclear cells (PBMCs) from women; Supplementary Figure S4: Effect of HPV16_(L1)_ and IL-2 on the lympho-proliferation of the total T-cells from peripheral blood mononuclear cells (PBMCs) isolated from women; Supplementary Figure S5: Effect of IL-2 and HPV16_(L1)_ treatment on the expression levels of three membrane markers involved in T-cell activation, CD71, CD95 and CD28, in human peripheral blood mononuclear cells (PBMCs) retrieved from women; Supplementary Figure S6: Influence of fetal bovine serum (FBS) concentration on the confluence of the HPV-positive HeLa cancer cell line; Supplementary Figure S7: Kinetic proliferation related to the effect of MIM-2, -3 and -4 in a model of starved HPV-positive cervical cancer cells.

## Abbreviations

APOBEC3: apolipoprotein B mRNA-editing enzyme catalytic polypeptide-like 3; ATCC: American Type Culture Collection; CH: centesimal Hahnemannian; CIN: cervical intraepithelial neoplasia; COX-2: cyclooxygenase-2; CsA: cyclosporin A; Ct.: control; DMEM: Dulbecco’s Modified Eagle Medium; DNA: deoxyribonucleic acid; ECACC: European Collection of Authenticated Cell Cultures; EFS: Etablissement Français du Sang; ELISA: enzyme linked immunosorbent assay; FBS; fetal bovine serum; GM-CSF: granulocyte macrophage colony stimulating factor; HEPES: 4-(2-hydroxyethyl)-1-piperazineethanesulfonic acid; HLA: human leukocyte antigen; HPV: human papillomavirus; HPV 16-L1 capsid-protein: HPV16_(L1)_; hr: human recombinant; HR: high-risk; IARC: International Agency for Research on Cancer; IFITMs: interferon-induced transmembrane proteins; IFN: interferon; IL: interleukin; IP-10: IFN-γ inducible protein of 10 kDa; IL-1Ra: IL-1 receptor antagonist; JAK1/2: Janus kinase ½; LD: low doses; MEM: minimum essential medium; MFI: median fluorescence intensity; MI: micro-immunotherapy; MIM: micro-immunotherapy medicine; MIP-1α: macrophage inflammatory protein-1α; PBMCs: peripheral blood mononuclear cells; PGE2: prostaglandin E2; pRb: retinoblastoma; RCU: read-copy update; RNA: ribonucleic acid; RNS: reactive nitrogen species; ROS: reactive oxygen species; RPMI: Roswell Park Memorial Institute; S.E.M.: standard error of the mean; S.D.: standard deviation; SN: supernatant; SNA: specific nucleic acid; STAT1: signal transducer and activator of transcription 1; Th: T-helper cell; TLR7: toll like receptor 7; TNF-α: tumor necrosis factor α; Treg: regulatory T-cell; TRIM: tripartite motif; ULD: ultra-low doses; Veh.: vehicle.
