# Supplementary material for: The Micro-Immunotherapy Medicine 2LPAPI® Displays Immune-Modulatory Effects in a Model of Human Papillomavirus Type-16 L1-Protein Capsid-Treated Human Peripheral Blood Mononuclear Cells and Antiproliferative Effects in a Model of Cervical Cancer Cells
Source: Cancers (Basel). 2024 Apr 5;16(7):1421. doi: 10.3390/cancers16071421 (PMC11010933; doi:10.3390/cancers16071421)
Supplement: Supplementary file 1 [file cancers-16-01421-s001.zip › cancers-2927602-supplementary.pdf]

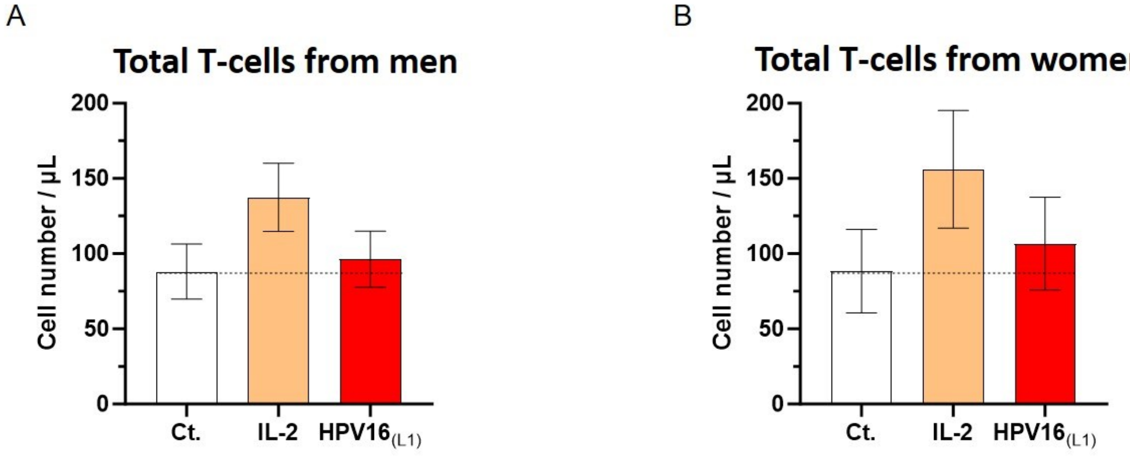

Figure S1. Influence of the L1-protein capsid of HPV 16 ($HPV16_{(L1)}$) on the proliferation of human peripheral blood mononuclear cells (PBMCs) isolated from (**A**) men and (**B**) women donors. Lympho-proliferation in the untreated control condition (Ct.), in the 20 ng/mL IL-2-treated condition, and in the $HPV16_{(L1)}$ (1/400 v/v)-treated condition. Data are illustrated as the mean ± standard error of the mean (S.E.M.) of the lympho-proliferation, in cell/µL for n = 4 replicates for each donor. The dotted line is drawn to highlight the effect of each treatment in comparison with the untreated Ct.

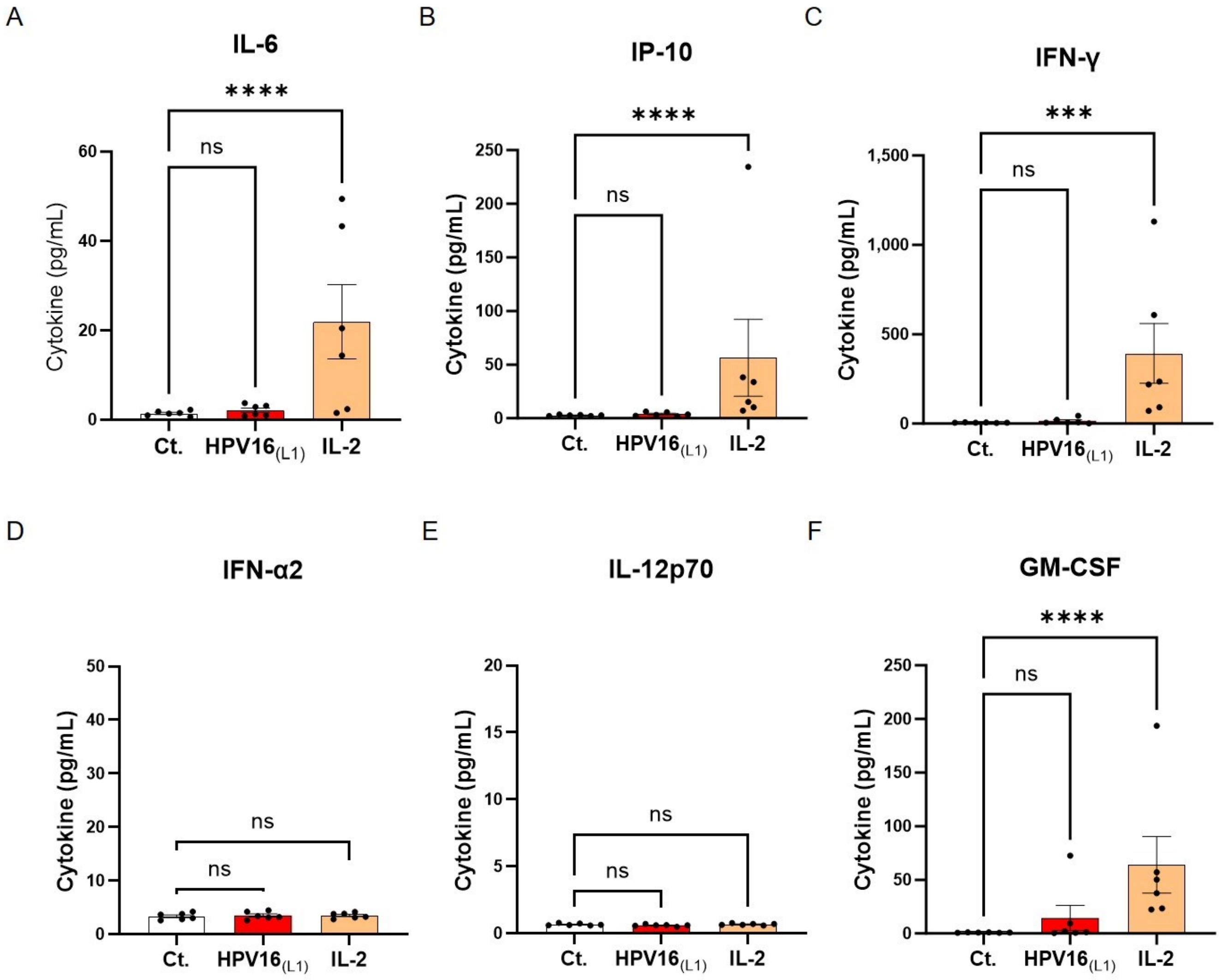

Figure S2. Effect of HPV16(L1) and IL-2 treatments on the secretion of cytokines involved in the anti-viral response in peripheral blood mononuclear cells (PBMCs) retrieved from women. The secretion of: (**A**) IL-6, (**B**) IP-10, (**C**) IFN-γ, (**D**) IFN-α2, (**E**) IL-12p70, and (**F**) GM-CSF, has been assessed by enzyme linked immunosorbent assay (ELISA). The secretion levels of these cytokines have been assessed in the control condition (Ct.; white histograms), or after treatment with either HPV16(L1) peptide pool (1/450 v/v; red histograms), or 20 ng/mL of IL-2 (orange histograms), within the super-natants (SNs) recovered from PBMCs isolated from women. Data are illustrated as the mean con-centrations (in pg/mL) ± S.E.M., obtained for n = 6 donors, in each treatment condition. Two-way ANOVA: ****p<0.0001, ***p<0.001, ns: non-significant.

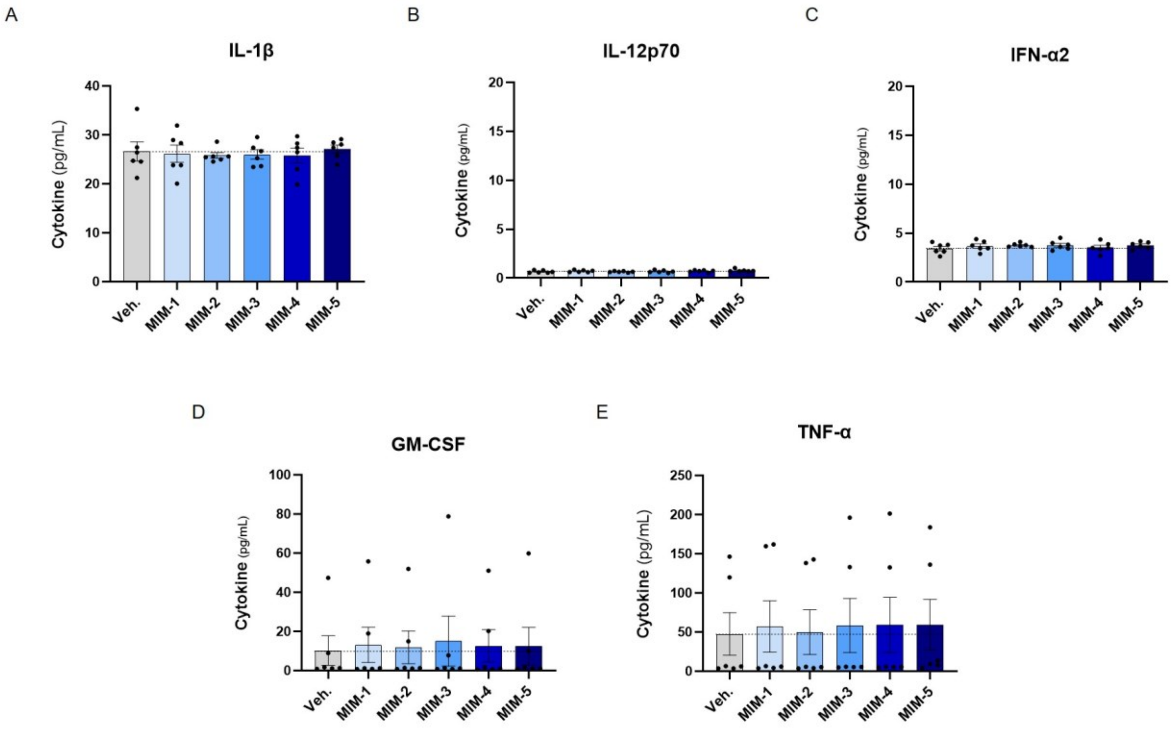

Figure S3. Effect of 2LPAPI on the secretion of cytokines involved in the anti-viral response in a model of HPV16(L1)-treated peripheral blood mononuclear cells (PBMCs) from women. The secretion of: (**A**) IL-1β, (**B**) IL-12p70, (**C**) IFN-α2, (**D**) GM-CSF, and (**E**) TNF-α, has been assessed by enzyme linked immunosorbent assay (ELISA) after treatment with either the vehicle (Veh.), MIM-1; -2; -3; -4 or -5, in the presence of HPV16(L1) (1/450 v/v), within the supernatants (SNs) recovered from PBMCs isolated from women. Data are illustrated as the mean concentrations (in pg/mL) ± S.E.M., obtained for *n* = 6 donors, in each treatment condition. The dotted lines are drawn to highlight the effect of each treatment in comparison with the Veh. condition.

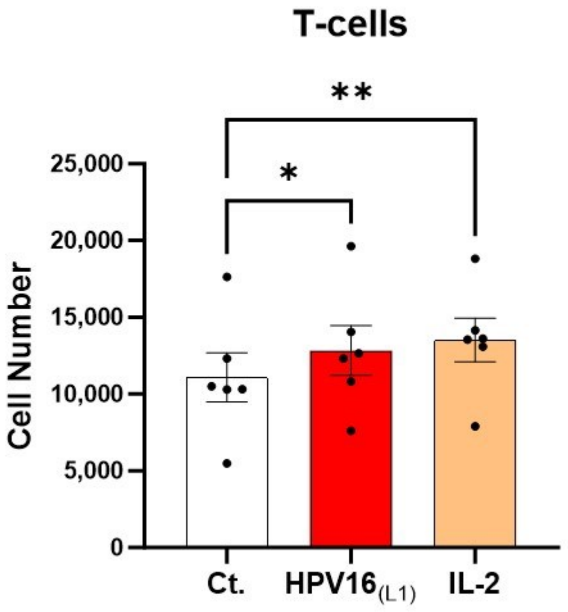

Figure S4. Effect of HPV16(L1) and IL-2 on the lympho-proliferation of the total T-cells from periph-eral blood mononuclear cells (PBMCs) isolated from women. Lympho-proliferation in the untreated

control condition (Ct.; white histogram), in HPV16(L1) (1/450 [v/v])-treated condition (red histogram), and in 20 ng/mL IL-2-stimulated condition (orange histogram). Data are illustrated as the mean cell number ± S.E.M., obtained for $n = 6$ donors, in each treatment condition. Each dot represents the mean value of the data collected from $n = 2$ replicate per donor. Two-way ANOVA: **$p<0.01$, *$p<0.05$.

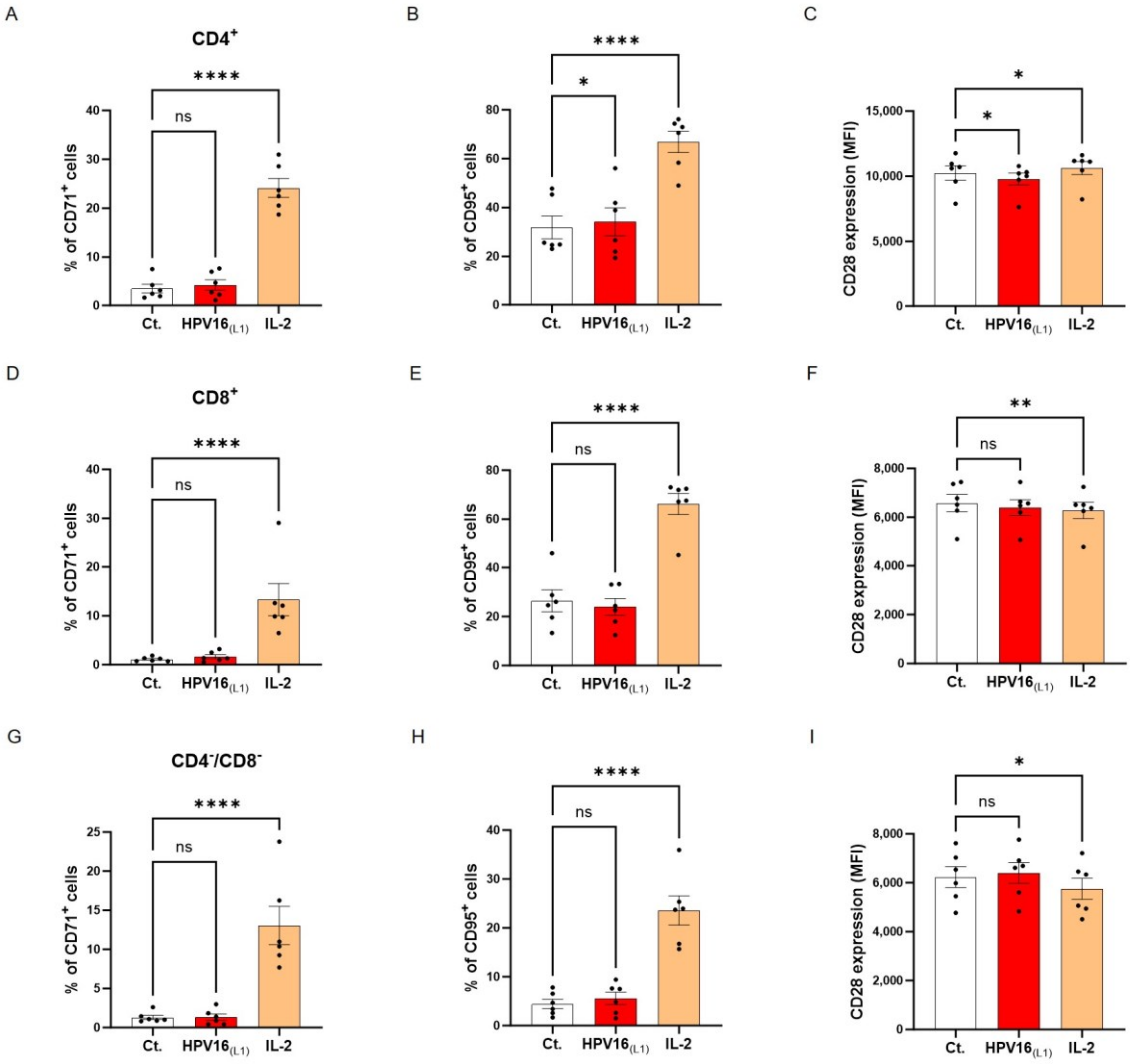

Figure S5. Effect of IL-2 and HPV16(L1) treatment on the expression levels of three membrane mark-ers involved in T-cells activation, CD71, CD95 and CD28, in human peripheral blood mononuclear cells (PBMCs) retrieved from women. The expression of these markers has been assessed by flow cytometry, in control condition (Ct.; white histograms), or after treatment with either HPV16(L1) pep-tide pool (1/450 [v/v]; red histograms), or 20 ng/mL of IL-2 (orange histograms), within the $CD4^+$ (**A-C**), $CD8^+$ (**D-F**), and $CD4^-/CD8^-$ (**G-I**) T-cells sub-populations. In (**A-B, D-E, G-H**), data are illustrated as the mean percentage of positive cells ± S.E.M., obtained for $n = 6$ donors, in each treatment con-dition. Regarding the expression of CD28, the results are expressed as the mean ± S.E.M. of the median fluorescence intensity (MFI) of the data collected from $n = 6$ donors, in each treatment con-dition (**C, F, I**). Two-way ANOVA: ****$p<0.0001$, **$p<0.01$, *$p<0.05$, ns: non-significant.

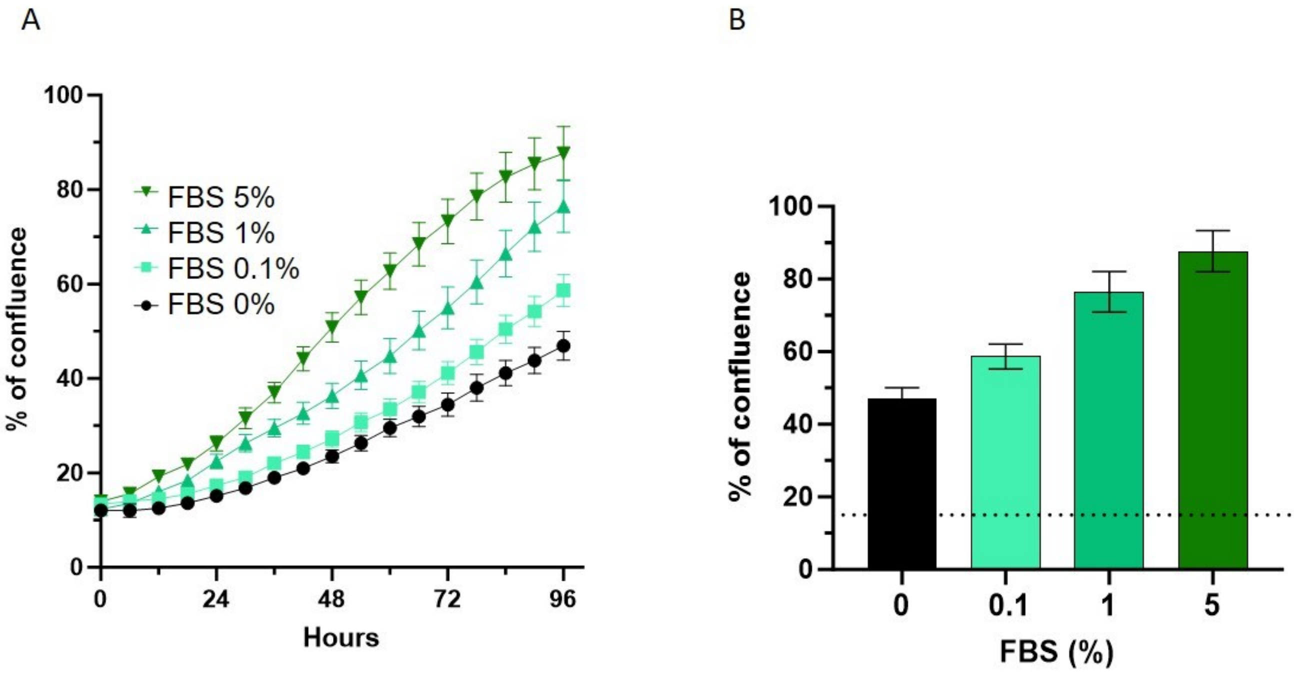

Figure S6. Influence of fetal bovine serum (FBS) concentration on the confluence of the HPV-positive HeLa cancer cell line. (**A**) Kinetics of HeLa cells confluence at different FBS concentrations (0%, 0.1%, 1%, and 5% [v/v]), measured by live content cell imaging system Incucyte HD, every 6 hours, over a total incubation time of 4 days in culture. The data is presented as the mean percentage of conflu-ence ± standard deviation (S.D.) for *n* = 3 replicates per condition. (**B**) Effect of different FBS concen-trations in the same conditions as presented in (**A**), on HeLa cells confluence after 4 days in culture. The data is presented as the mean percentage of confluence ± S.D. for *n* = 3 replicates per condition. The dotted line in (**B**) highlights the percentage of confluence on Day 0, for each condition.

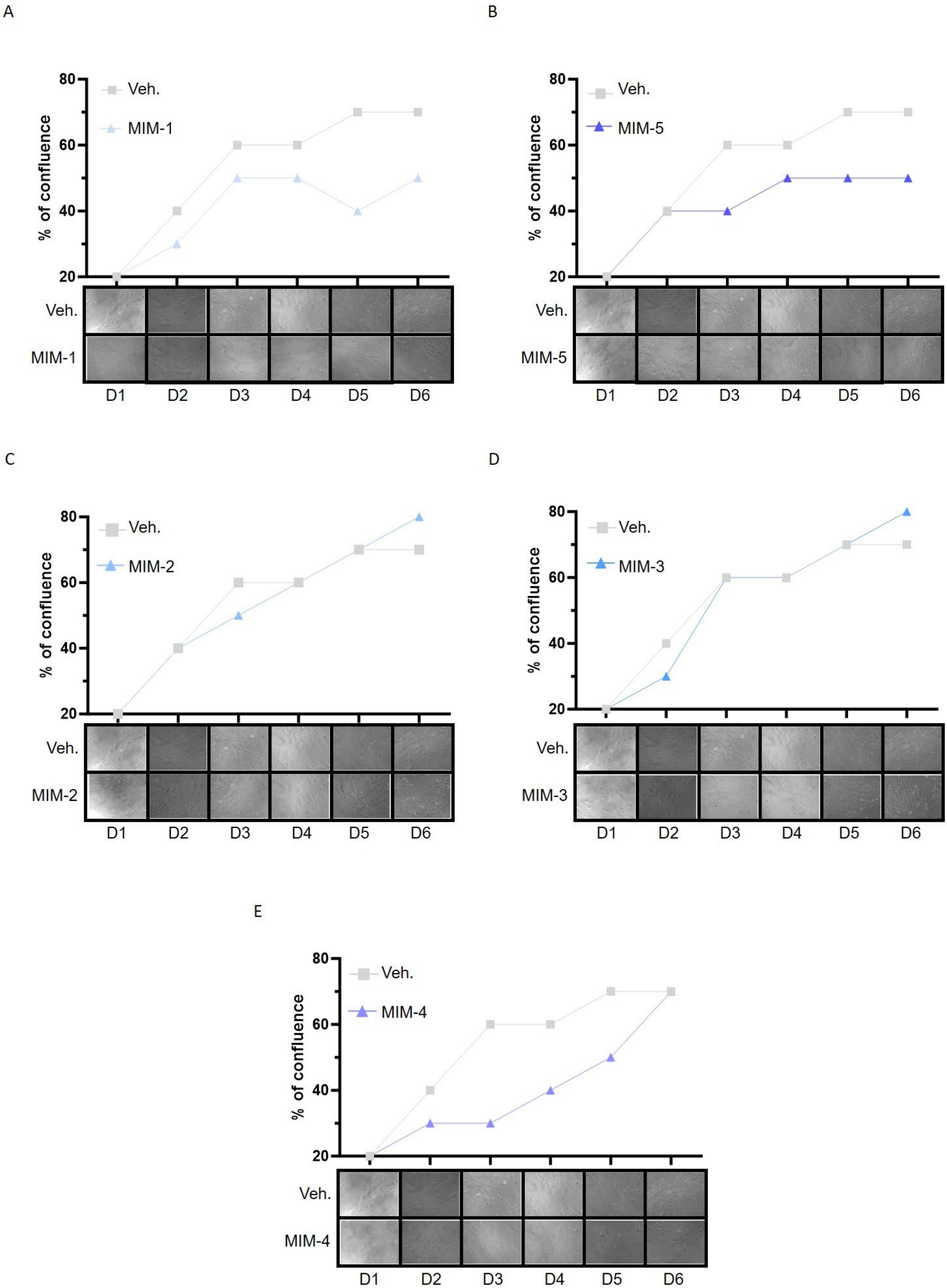
A
80
60
40
20
% of confluence
Veh.
MIM-1
Veh.
MIM-1
D1
D2
D3
D4
D5
D6
B
80
60
40
20
% of confluence
Veh.
MIM-5
Veh.
MIM-5
D1
D2
D3
D4
D5
D6
C
80
60
40
20
% of confluence
Veh.
MIM-2
Veh.
MIM-2
D1
D2
D3
D4
D5
D6
D
80
60
40
20
% of confluence
Veh.
MIM-3
Veh.
MIM-3
D1
D2
D3
D4
D5
D6
E
80
60
40
20
% of confluence
Veh.
MIM-4
Veh.
MIM-4
D1
D2
D3
D4
D5
D6

Figure S7. Kinetic proliferation related to the effect of MIM-1; -2; -3; -4 and -5 in a model of starved HPV-positive cervical cancer cells. HeLa cells were treated with either the vehicle (Veh.), MIM-1 (**A**), MIM-5 (**B**), MIM-2 (**C**), MIM-3 (**D**), or MIM-4 (**E**), for five consecutive days (D) (from D2 to D6, as detailed in section **2.3.** of Materials & Methods**,** see **Figure 1**). The cell confluence has been visually estimated by microscopy, and the results are presented, each day, from Day 1 (D1), to D6, for the observation of one replicate per condition. Representative pictures of the wells were taken by mi-croscopy, and are shown under the corresponding timepoints, for each treatment condition, using a 20X magnification.
